# Cognitive Training With Head-Mounted Display Virtual Reality in Neurorehabilitation: Pilot Randomized Controlled Trial

**DOI:** 10.2196/45816

**Published:** 2023-07-21

**Authors:** Julian Specht, Barbara Stegmann, Hanna Gross, Karsten Krakow

**Affiliations:** 1 SRH University of Applied Sciences Heidelberg Department of Applied Psychology Heidelberg Germany; 2 Asklepios Neurologische Klinik Falkenstein Department of Neurorehabilitation Königstein im Taunus Germany; 3 Rehaklinik Zihlschlacht Department of Neurorehabilitation Zihlschlacht Switzerland

**Keywords:** cognitive rehabilitation, virtual reality, neurorehabilitation, psychology, stroke

## Abstract

**Background:**

Neurological rehabilitation is technologically evolving rapidly, resulting in new treatments for patients. Stroke, one of the most prevalent conditions in neurorehabilitation, has been a particular focus in recent years. However, patients often need help with physical and cognitive constraints, whereby the cognitive domain in neurorehabilitation does not technologically exploit existing potential. Usually, cognitive rehabilitation is performed with pen and paper or on a computer, which leads to limitations in preparation for activities of daily living. Technologies such as virtual reality (VR) can bridge this gap.

**Objective:**

This pilot study investigated the use of immersive VR in cognitive rehabilitation for patients undergoing inpatient neurorehabilitation. The goal was to determine the difference in rehabilitation effectiveness between a VR serious game that combines everyday activities with cognitive paradigms and conventional computerized cognitive training. We hypothesized the superiority of the VR serious game regarding cognitive abilities and patient-reported outcomes as well as transfer to daily life.

**Methods:**

We recruited 42 patients with acute brain affection from a German neurorehabilitation clinic in inpatient care with a Mini Mental Status Test score >20 to participate in this randomized controlled trial. Participants were randomly assigned to 2 groups, with 1 receiving the experimental VR treatment (n=21). VR training consisted of daily life scenarios, for example, in a kitchen, focusing on treating executive functions such as planning and problem-solving. The control group (n=21) received conventional computerized cognitive training. Each participant received a minimum of 18 treatment sessions in their respective group. Patients were tested for cognitive status, subjective health, and quality of life before and after the intervention (Alters-Konzentrations-Test, Wechsler Memory Scale—Revised, Trail Making Test A and B, Tower of London—German version, Short Form 36, European Quality of Life 5 Dimensions visual analog scale, and Fragebogen zur Erfassung der Performance in VR).

**Results:**

Repeated-measures ANOVA revealed several significant main effects in the cognitive tests: Tower of London—German version (*P*=.046), Trail Making Test A (*P*=.01), and Wechsler Memory Scale—Revised (*P*=.006). However, post hoc tests revealed that the VR group showed significant improvement in the planning, executive control, and problem-solving domains (*P*=.046, Bonferroni *P*=.02). In contrast, no significant improvement in the control group between *t_0_* and *t_1_* was detected (all *P*>.05). Furthermore, a nonsignificant trend was observed in visual speed in the VR group (*P*=.09, Bonferroni *P*=.02).

**Conclusions:**

The results of this pilot randomized controlled trial showed that immersive VR training in cognitive rehabilitation had greater effectiveness than the standard of care in treating patients experiencing stroke in some cognitive domains . These findings support the further use and study of VR training incorporating activities of daily living in other neurological disorders involving cognitive dysfunction.

**Trial Registration:**

Federal Registry of Clinical Trials of Germany (DRKS) DRKS00023605; https://drks.de/search/de/trial/DRKS00023605

## Introduction

### Background

The primary goal of neurorehabilitation is to restore or preserve critical function, thereby enabling patients to participate in daily life. Neurological diseases can lead to severe disabilities. This requires the patient to make great effort in rehabilitation. For example, stroke is one of the leading causes of disability. It is estimated that 33% to 42% of stroke survivors require assistance in managing activities of daily living (ADL). Reduced ability to manage ADL is often long term, with 20% of patients being unable to manage ADL independently 5 years after a stroke [1,2]. Even patients with only mild stroke have unmet rehabilitation needs and require support in managing ADL [3,4]. This also raises the question of whether there should be more ADL-focused therapies in principle, because the study by Rejnö et al [1] showed that there is a continuous decrease in ADL independence over time after a stroke; the proportion of patients who can independently manage ADL 3 months after a stroke decreases by about 3% per year, so that 5 years after the stroke, only just over 80% of the group of independent patients can independently manage ADL [1].

Furthermore, stroke is associated with a substantial economic burden, creating a cost of €60 billion (US $67.2 billion) in the European Union [5]. Wilkins et al [6] highlighted that these costs are far greater when including indirect costs from the subsequent burden on society by lost employment and expenses related to the need for multidisciplinary, extensive rehabilitation. This points to the great need for more effective rehabilitation interventions that support patients in regaining their ability to participate and manage everyday life independently.

ADL, such as making coffee or preparing lunch, require multiple motor and cognitive processes to achieve their functional goals. Seemingly trivial activities demand several temporally overlapping cognitive functions such as planning, problem-solving, recall, and execution [7]. Toth et al [8] demonstrated in collaboration with 36 occupational therapists that the 20 most critical instrumental ADL were closely related to different cognitive functions in older adults. Cognitive domains that have been frequently discovered in this context are memory, executive functions (EF), attention, visuospatial abilities, and language. They also found that, for instance, in grocery shopping, making and keeping medical appointments and medication management EF were the most demanded cognitive domains, whereas paying bills, making a phone call, and making and keeping medical appointments were identified to require language the most [8]. In addition, there exists a correlation between ADL performance, multimorbidity, and quality of life [9]. Therefore, it would be beneficial if neurorehabilitation methods emphasized preparation for everyday life to reduce morbidity. One component of this process is improving the cognitive skills required to handle ADL. With the findings of Toth et al [8], it would be beneficial if rehabilitation explicitly addresses patients’ requirements in everyday life and provides training, especially on the more complex instrumental ADL and its cognitive functional domains.

However, computerized cognitive training (CCT), or pen-and-paper training, is the standard practice in German rehabilitation facilities. It involves training in isolated domains, including attention, working memory, EF, and language. They are economical and efficient tools for delivering therapies. Still, its use is recommended only with restrictions by national guidelines for treating EF in various diseases, for example, in the stroke guidelines [10].

Furthermore, researchers discovered insufficient evidence of computer-based training in a second-order meta-analysis in neurorehabilitation [11]. The authors concluded that the processes initiated by CCT are often limited to near-transfer effects. The effects remained within the scope of the trained task regardless of the far-transfer measure, study population, or cognitive training application used. However, a far-transfer effect is crucial for later-stage rehabilitation and return to daily life. This report aligns with the above-referenced stroke guidelines, advising caution using computer-based training owing to the limited evidence on its effectiveness. Although Gajewski et al [12] demonstrated in their clinical trial that some far-transfer effect occurs when conducting combined PC and paper-and-pencil training, a transfer effect on ADL was not detectable.

Instead, customized virtual reality (VR) training is suitable for multidomain ADL training [13,14]. VR is defined as images and sounds created by a computer that seem almost indistinguishable from the real world and users can interact with via sensors [15]. Although it can be experienced nowadays with purpose-made head-mounted displays (HMDs) placed on the head in front of the eyes for a fully immersive experience, the interaction with the virtual world is mainly carried out with 2 hand controllers. VR enables a combination of principles from occupational therapy, neuropsychology, and ADL, resulting in an ecologically valid training setting for many individuals with neurological impairments who want to regain their ability to participate in daily activities. Although insights exist concerning the acceptance of immersive HMD-VR in patients with stroke [16] or older adults [17], knowledge about the processes of cognitive rehabilitation facilitated by immersive VR needs to be expanded.

One concept that is becoming more widespread in neurorehabilitation is serious games (SG) [18]. SGs are defined as games developed not only for entertainment but also for serious learning purposes and can be considered a nonpharmacological, gamified approach to treat and evaluate [19] a patient’s executive dysfunction in an ecologically valid way [20]. SGs and VR have already been combined, as Faria et al [21] demonstrated. They trained EF in immersive VR with a virtual city, Reh@City, including attention, memory, visuospatial abilities and other EF, in performing ADL and found that this approach boosts cognitive rehabilitation and leads to better results in contrast to conventional cognitive rehabilitation methods [21]. A systematic review of the assessment and treatment of EF with VR in clinical populations and healthy participants concluded that VR is a promising tool [22]; however, more research is necessary, as the authors cited only a few trials on this topic. Training systems focused on ADL have the potential for better transfer to the real world after rehabilitation. Still, more evidence on the interrelation between SGs, ADL, EF training, and immersive VR [22] is necessary.

Investigating VR-based cognitive rehabilitation involves research of a multitude of groups. The number of publications in scientific journals has increased almost exponentially since 2004 [23]. The 3 most frequently studied diseases in VR-based cognitive rehabilitation are stroke, dementia, and mild cognitive impairment. Jahn et al [13] noted in their systematic review of publications about VR and cognitive rehabilitation that there is promising evidence for the functionality of this approach. However, current research findings still struggle with several issues, including group sizes being too small (N=6-34 participants, with 89% of reviewed trials having N<20 participants per intervention arm), the lack of randomization (eg, no randomization at all), or suboptimal statistical analyses (no group-by-time interaction analysis reported).

Maggio et al [14] found in their review of VR for cognitive rehabilitation that VR training in neurorehabilitation has measurable effects on executive functioning, visuospatial abilities, speech, attention, and memory skills. In particular, executive functioning and depression at discharge from stroke rehabilitation are strong predictors of functioning at the time of discharge and the following 12 months, as Shea-Shumsky et al [24] found in their study that executive functioning was a strong predictor of ADL capacity to a more significant extent than mental status. It is a strong signal that problems with EF are involved in issues regarding functionality in everyday life and that viable training is helpful and necessary [24].

### Objectives

In this paper, we present the results of a pilot clinical randomized controlled trial in patients undergoing neurological treatment after acute brain injury. The trial compared two treatments: (1) a novel immersive VR intervention to treat cognitive dysfunction, specifically EF, presenting ADL in the form of an SG, and (2) conventional computerized cognitive rehabilitation training in inpatient care at a German rehabilitation hospital. VR intervention includes training in a virtual kitchen or garden, simultaneously targeting various cognitive domains, including planning, attention, and problem-solving. Furthermore, interaction with the virtual environment was enabled using HMD-VR to fully immerse users. The trial was conducted to investigate whether training with a VR-SG produces measurable effects in different cognitive domains in patients after acute brain injury and whether the potential effects outperform, are similar to, or are inferior to traditional training methods, specifically CCT.

We hypothesized that VR training will significantly improve cognitive performance and outcomes related to quality of life and general health compared with our control condition throughout the 4-week treatment. Therefore, this study addressed the following objectives:

- The primary objective was to assess the impact of an ADL-based VR-SG on patients’ cognitive abilities after acute brain injury throughout a 4-week treatment period and compare it with a conventional CCT treatment.
- The secondary objective was to assess the impact of an ADL-based VR-SG on self-reported outcomes, quality of life, state of health, and affect and compare it with conventional CCT treatment.
- The tertiary objective was to assess the impact of an ADL-based VR-SG on the actual transfer of learned abilities in VR to daily life and reality compared with conventional CCT treatment.
- The exploratory objective was to investigate the extent to which an ADL-based VR-SG is suitable for cognitive rehabilitation from phase C onward.

No changes to these objectives were made after trial commencement.

## Methods

### Participants

Participants were screened and recruited for enrollment at the Asklepios Neurologische Klinik Falkenstein clinic in Königstein im Taunus, Germany. The patients were transferred to the rehabilitation clinic directly from the emergency hospital (rehabilitation phase A) or had already undergone early rehabilitation (rehabilitation phase B). The differentiation to which unit a patient was admitted was based on the Barthel index: it was first developed by Mahoney and Barthel in 1965 [25] and Colin et al [26] and Shah et al [27] modified it over the years. The original 10-item form consists of 10 scales describing ADL, including feeding, bathing, grooming, dressing, bowel and bladder control, toilet use, transfers (bed to chair and back), mobility, and stair climbing. The rating describes the independence of patients to perform the named ADL on a scale from 0 to 10, with a maximum of 15 points for the transfers and mobility scale. The maximum score was calculated by summing up the results of all the scales. A maximum score of 100 indicates that patients have no difficulty managing the described ADL, whereas a score between 30 and 70 indicates the patient’s ability to conduct rehabilitation phase C in Germany. All patients were admitted to the rehabilitation phase C unit and had subacute stroke syndromes. The trial was planned using a parallel design.

The admission interview at the rehabilitation clinic and records of newly admitted patients undergoing rehabilitation were used to recruit the sample. A phase C status qualified patients to participate in the clinical trial, and a transition to phase D during study participation was possible. None of the patients started earlier or later than phase C. All participants were required to be legally competent and of age and had to undergo inpatient neurological rehabilitation treatment at the neurorehabilitation clinic because of acute brain affection (eg, cerebral infarction, intracerebral hemorrhage, subarachnoid hemorrhage, subdural or epidural intracranial hemorrhage, traumatic brain injury, brain tumor, meningitis or encephalitis, and hypoxic encephalopathy), had to have full functionality for at least one arm, had known adequate or corrected eyesight in the central field of vision, and had to give written consent to participate. Patients were also screened for cognitive performance using the Mini Mental Status Test (MMST) [28]. The score obtained had to be >20 out of 30 points. The cut-off value was chosen because some cognitive impairment was expected in this sample, and scores of 27 to 30 (no cognitive impairment) would not properly represent the sample of patients undergoing neurorehabilitation. Patients were excluded if they had present or prior psychiatric comorbidities or disorders and a known overreaction to visual stimulation leading to seizures. Once patients eligible for the study were identified, they were targeted by trained study personnel and informed of the opportunity to participate. In this course, the content and objectives of the study are explained.

### Materials: Training Tools

**Figure 1:**
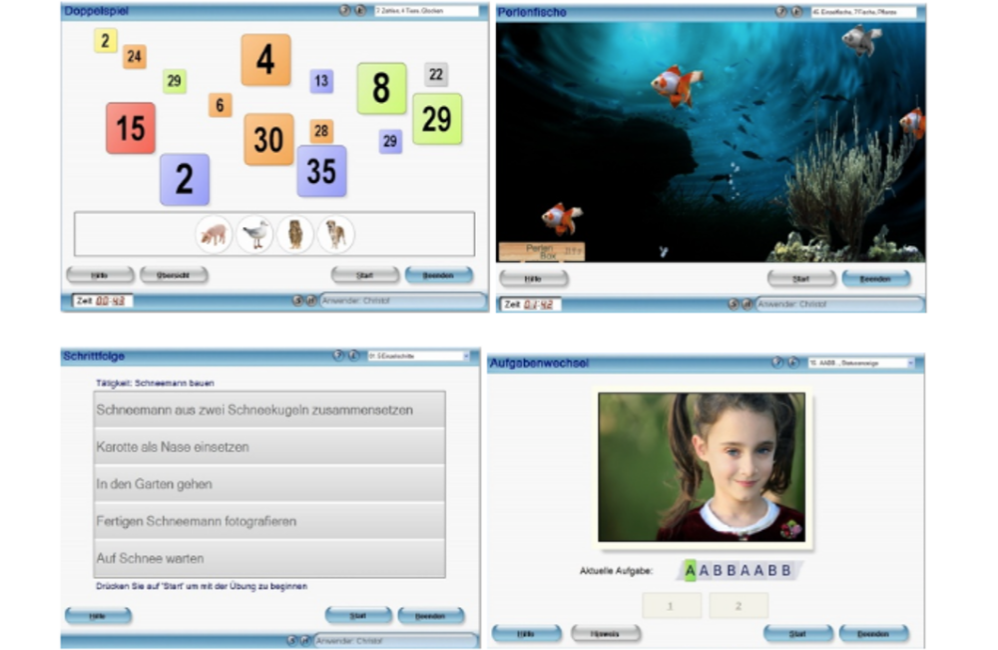
Computerized cognitive training exercises, representing the exercises of Freshminder; upper images: Doppelspiel (double play) and Perlenfische (pearlfish), bottom images: Schrittfolge (step sequence) and Aufgabenwechsel (tasks switching).

For the trial, 2 different treatment methods were applied. The control group received CCT used in routine care at the rehabilitation center at least 3 times a week for 30 minutes each session, in this case, Freshminder. Freshminder is a cognitive training program with multiple training paradigms, focusing on attention, planning, and memory (Figure 1). Training was conducted on a PC with a keyboard and mouse. Users must interact with the application by clicking objects or deciding by pressing a key on the keyboard. In this study, the following tasks were considered: pearlfish (vigilance and working memory), picture series (memory), step sequence (action planning), task switching (flexibility), and double play (divided attention). For example, in the pearlfish task, participants had to click on fish carrying pearls. Participants had to discriminate which fish were holding the pearls and identify the fish at the correct time so that the pearl fell appropriately into the box at the bottom of the sea. This exercise trained selective attention, visual perception, and visuomotor skills. All exercises were adapted to the patient’s performance by changing the difficulty and level at the beginning of each task. This was adapted by the therapist in charge. Patients received information about their performance during the training tasks. They could choose from a subset of exercises with the therapist, ensuring that all exercises were performed at a well-balanced frequency.

The VR group received treatment with an early version of Teora mind, conceptualized and developed by the medical device manufacturer Living Brain, a cognitive training in immersive VR. Teora mind is a certified medical device of risk class IIa according to Medical Device Regulation (EU) 2017/745. The certification procedure was executed by Notified Body TÜV Süd in 2021. The SG integrates ADL and cognitive training methods in a playful way to improve cognition and transfer into daily life and consists of realistic scenarios for training specific abilities.

**Figure 2:**
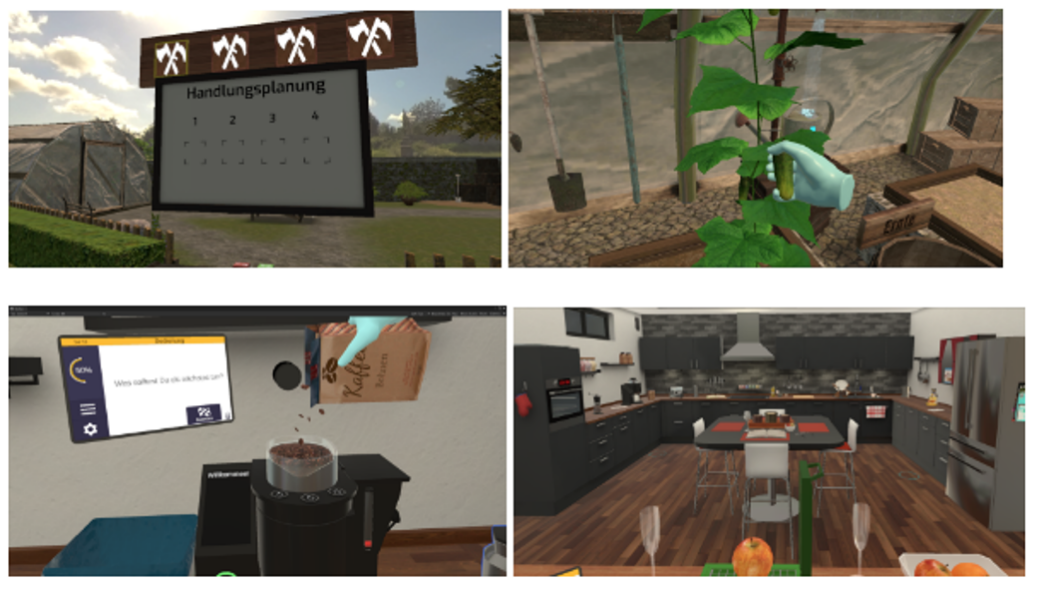
Virtual reality training scenarios, upper images: virtual garden scenario, showing action planning board (left) and greenhouse with user harvesting cucumbers (right); bottom images: virtual kitchen scenario, showing user refilling coffee machine with coffee beans (left) and start screen when entering the kitchen (right).

The participants were trained in a virtual garden and kitchen scenario. Patients were asked to make coffee for multiple people in the kitchen. Therefore, they had to actively implement all the necessary steps for serving coffee, including filling the coffee machine with beans and water, changing the coffee filter, choosing a mug, and considering whether the coffee had to be served with milk and sugar (Figure 2). Other exercises included sorting groceries according to their categories, for example, beverages, fruits and vegetables, and durable food for stock, or sorting out the refrigerator according to specifications, such as spoiled food. A time pressure component was added with increasing difficulty. All activities were implemented in real time and through patient interactions with the virtual environment.

In the garden scenario, patients were asked to plant seeds to grow strawberries, tomatoes, and cucumbers. In the growth phase, users had to ensure that there was always sufficient water in the bed and that popping weeds did not destroy the seedlings, which required watering the bed with a watering can. After successfully growing fruits and vegetables, users can produce juices from the harvested food. Participants had to plan their activity by first sorting picture cards describing each action step on a board in what they thought was the correct order. Next, they were required to confirm their orders. Finally, the system reported whether the order was correct or incorrect and indicated the steps that were arranged incorrectly. The exercise could begin only after the right order was achieved. The complexity varied depending on the difficulty level; at the highest level, planning was omitted and the activity had to be started immediately.

The main cognitive domains targeted by this VR training are EF, in particular action planning, problem-solving, selective attention, and working memory. The specific pieces of training themselves do not primarily focus on a single cognitive domain but rather on training the ADL, which typically comprises multiple cognitive domains simultaneously. Similar to the CCT intervention, the difficulty could be adapted to individual performance by changing to 20 different degrees of difficulty, which was adjusted by the therapist in charge. Patients received information on how they performed their training tasks in the VR application. In this trial, an early version, specifically designed for this clinical trial, was used. At that time, the medical device had not been introduced to the market. It was updated 2 times during the trial for minor bugs.

VR devices of the type Oculus Quest (manufacturer: Meta) were used for the trial conduction. The device is a stand-alone HMD, and additional technical devices for experiencing immersive VR are not required. In addition to the VR device, the study nurses and therapists in charge received a tablet (Samsung Galaxy Tab A) for monitoring ongoing VR activities; all VR procedures were mirrored on the tablet in real time. This allowed patient-therapist interaction despite the high grade of immersion, particularly in the first training sessions of benefit. Interaction in immersive VR was enabled by the hand controller used by the patients. The blue hands visually represented them and were congruent with the actual movement of the upper extremities. The controllers included multiple buttons, of which only 2 were necessary to use the VR software. Moving the head makes it possible to gain a complete overview of the virtual world. Through a visible white beam, users can assess what they are currently pointing at in a virtual environment. This allowed for the selection of objects. To introduce the patients to the technology and virtual environment, they went through a dedicated tutorial at the beginning of each treatment. High resolution pictures of the training are available in Multimedia Appendices 1-4.

### Procedure

**Figure 3:**
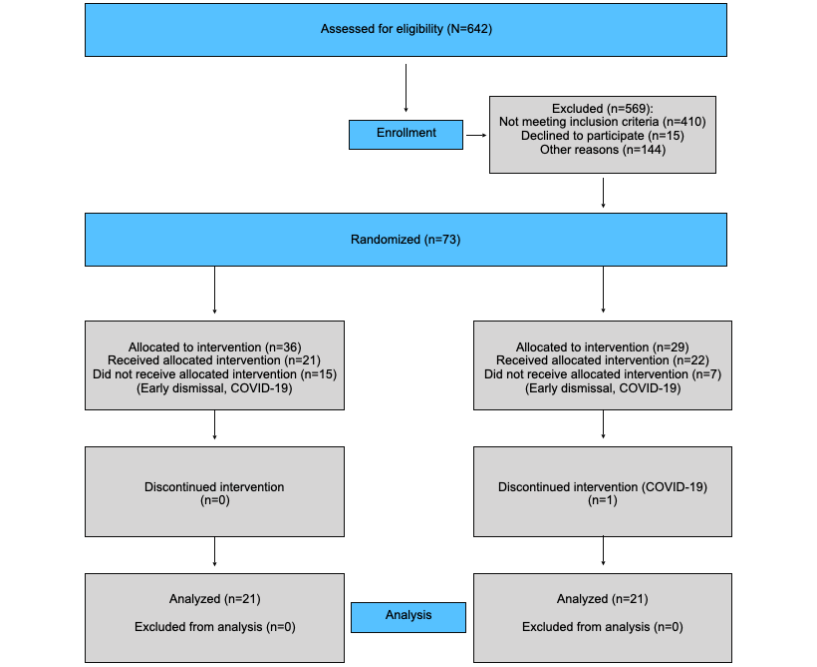
CONSORT (Consolidated Standards of Reporting Trials) flow diagram.

In the first 2 sessions, which lasted approximately 60 minutes each, comprehensive testing (Alters-Konzentrations-Test [AKT], Wechsler Memory Scale—Revised [WMS-R], Trail Making Test [TMT] A and B, Tower of London—German version [TL-D], Short Form 36 [SF-36], visual analog scale [VAS; EQ-5D VAS], and Fragebogen zur Erfassung der Performance in VR [FEPVR]) was conducted (*t_0_*). The testing consisted of psychological tests conducted by a psychologist and self-assessed questionnaires. After completing the testing, individuals were randomly assigned to 1 of the 2 training groups by drawing from an urn, which was performed by the study nurse. Only the responsible investigator had access to the list of individuals in each group. Because of the nature of the study, it was impossible to ensure the blindness of the investigator responsible for the intervention and participants. The process of participant randomization is described in the flowchart prepared according to the CONSORT (Consolidated Standards of Reporting Trials) guidelines (Figure 3), and we used an analog randomization process by drawing notes from an urn. The whole CONSORT checklist is available in Multimedia Appendix 5. Simple randomization was performed by the study nurse. The treatment was scheduled for 18 to 25 sessions with a duration of 30 minutes for the individual, based on the date of admission and the participant’s immediate health and neurological condition. The sessions were conducted at least 3 to 5 days per week. Both groups received their allocated treatments from day 3 to day 20. A maximum of 25 treatment sessions were allowed.

The study nurse instructed the intervention group on the device’s functions and use before the first VR training session. This included the general operation of the device from the outside with the attached buttons (on-off button and volume control), especially the handling of the hand controllers. After receiving the instructions, participants went through a VR tutorial, consisting of training in basic functionality, a memory game, and a recycling game, in which they were familiarized with general handling in the first training session. This tutorial demonstrated the interaction with the VR environment, virtual objects, and the handling of the entire experience. The tutorial was developed and tested with stroke patients, leading to high acceptance in a group of patients with stroke and healthy older adults, indicating that it is suitable for introducing this critical user group to immersive VR [16]. In addition, the familiarization phase helps reduce possible effects due to unawareness about the technology and negative attitudes toward it [29]. During all training sessions, the study nurse was able to track the process on a tablet and was thus able to provide accurate, customized assistance if needed.

The control group was also instructed on the operation of the used computer if needed, and an introduction to the program, explaining the different tasks, before the first training session was conducted by the study nurse in charge. During all training sessions, the study nurse was close to the participant and was thus able to provide accurate, customized assistance if needed.

The patients remained statically seated in the same place throughout all training sessions to minimize the potential risks of injury. Locomotion was performed using only a controller in a virtual environment. The study nurse documented the task results during the training sessions (ie, the time needed to finish a task and other observations), in a separate, pseudonymized document. The patients could choose from the exercises themselves, with the therapist ensuring that all exercises were performed at a well-balanced frequency. All participants could stop the treatment at any time, for example, if they felt unwell during training.

Immediately after the seventh training session, participants in both groups were asked to complete the Positive and Negative Affect Schedule (PANAS) questionnaire and rate their current subjective health status using the EQ-5D VAS. After the final treatment session, the test battery was repeated (*t_1_*). Furthermore, a survey regarding the overall VR experience was conducted using open-ended questions, and the patient was asked for suggestions for improvement and satisfaction with the application.

### Dependent Measures

#### Overview

Each participant was comprehensively tested at 2 points: before treatment (*t_0_*) and immediately after the last session (*t_1_*). The focus was on investigating the impact of the 2 cognitive training methods on diverse cognitive abilities. We tested the patients regarding working memory, selective attention, planning, attention, task organization, cognitive flexibility, and problem-solving. We also examined their quality of life, affect, and health status. In addition, a new measurement tool was tested to evaluate subjective performance in a situation similar to VR training to determine the extent to which the VR training was transferred to reality. The test battery included the following tests.

#### AKT Measure

The AKT [30] is a psychometric procedure that measures concentration ability and vigilance. The test was developed and standardized specifically for older people and is therefore adapted to their needs in terms of comprehensibility, task difficulty, and resilience requirements. Participants had to differentiate a figure at the top of the test sheet from a series of similar figures and cross it out. In this trial, we used the German version.

#### TMT A and B Measure

The TMT [31] is a cognitive test for measuring EF, particularly visuomotor abilities, which are essential for executing ADL properly [24]. In TMT A, patients had to connect numbers in ascending order; in TMT B, they had to connect numbers and letters in ascending and alternating order. The test has been standardized for patients with cognitive disorders undergoing neurological rehabilitation and is often used for neurorehabilitation screening and diagnostics.

#### TL-D Measure

The TL-D is a transformation task that captures convergent problem-solving thinking and planning processes [32]. The objective of the task is to transfer 3 different-colored balls from an initial state to a specified target state in a minimum number of moves. The test consists of 20 tasks of varying severity and has been standardized in a sample of patients with neurological diseases; we used the German version of the test.

#### WMS-R: Number Span Forward and Backward

The WMS-R measures different memory functions [33]. In this study, a subset number span was used. First, the therapist reads a series of numbers of increasing length out loud. Depending on the task, the patient was then asked to recall the numbers in the correct order, either forward or backward. The WMS-R has been tested and standardized in patients with neurological and psychiatric disorders.

#### SF-36 Measure

The SF-36 questionnaire assesses patients’ health-related quality of life and consists of 36 items [34]. It measures 8 dimensions of subjective health: physical functioning, physical role functioning, physical pain, general health perception, vitality, social functioning, emotional role functioning, and psychological well-being. The SF-36 has been standardized in various patient populations; we used the German version of the instrument in this trial.

#### EQ-5D VAS Measure

Scientists use EQ-5D instruments developed by EuroQol for many different diseases. The EQ-5D VAS is a VAS in the standard layout of a vertical 20-cm scale with a range of values from 0 to 100, asking respondents to rate their current health status on the scale. The higher the rating, the better the indicated health status. The tool has been used in more than 117 countries and standardized for multiple patient groups.

#### FEPVR Tool

The VR performance instrument is a measurement tool to assess how well everyday activities can be performed and what effects training in VR has on daily performance. The test consisted of questionnaires, individual actions that were actively performed, and observation forms for therapists to rate patient activities. These activities included, among others, actively sorting laundry, planting strawberries, self-assessment of the ability to plan an action, and quantitative and qualitative feedback on the training experience. Our research group developed this tool specifically for this trial; however, it has yet to be validated in a large clinical sample. Therefore, the use was experimental. Book et al [35] and Graessel et al [36] developed and published a similar testing tool.

#### PANAS Trial

For this trial, we used the German version of the PANAS [37]. This questionnaire measured positive and negative affective states and traits. The patient rated the intensity of a sensation or feeling on a 5-point scale from “not at all” to “extremely.” Twenty items were presented.

### Power and Statistical Analysis

Statistical analyses were performed using SPSS 29 (IBM Corp), with statistical significance set at *P*<.05. We performed repeated-measures ANOVA and post hoc tests to detect inner-subject and between-group changes between *t*_0_ and *t*_1_.

Comparing the VR and control groups in terms of cognitive performance and health-related measures involved group mean comparisons from 2 independent samples. Power calculations (power analysis tool GPower [Heinrich-Heine-Universität Düsseldorf, release 3.1.9.6 for Mac OS X]) revealed that our sample size of n=21 per group was sufficient to detect a standardized estimated population mean difference of *f*=0.25, which is considered a medium effect size, with a power of 1 – β = .88 – α = .05. This shows that our small sample size did not constrain the detection of between-group effects at a conventional power goal of 0.8 with a medium effect size of *f*=0.25.

### Ethics Approval

Approval for this pilot trial was obtained from the local Ethics Committee of the Regional Medical Association Hessen, Germany, under approval 2020-1768-fMP. The VR training Teora mind is a certified medical device (class IIa) in line with the European Medical Device Regulation and therefore obtained approval to conduct this trial from the state authority Bundesinstitut für Arzneimittel und Medizinprodukte under the file number 94208-5660-12543. All regulatory approvals were obtained before the recruitment of the first patient.

## Results

### Baseline Characteristics

The recruitment process started on November 1, 2020, and the last patient completed the treatment on March 31, 2022. We initially planned to include 52 patients but decided to close the study when we reached 42 patients finishing all treatment sessions because this sample size was large enough to calculate repeated-measures ANOVA. Further extension of the trial was not possible due to administrative reasons. A total of 21 patients received the novel VR treatment, whereas the other 21 received control CCT. Furthermore, 33% (14/42) of the patients were female. Patients in the control group were, on average, aged 67.3 (SD 4.6) years at trial participation, while the VR group had a mean age of 68.3 (SD 14.5) years at trial participation; no statistical difference between both groups regarding age was found (*P*=.69). Detailed information on the brain damage of the included participants is found in Table 1. For more detailed information on the demographic characteristics, mean performance, and SD for all sample measures in this clinical trial, please refer to Tables 2 and 3.

Of the 642 patients receiving treatment at the center at the time of trial conduction, 73 patients were primarily included for participation, of which 42 (14 female patients) completed all treatment sessions. However, 31 patients were not able to complete the minimum treatment number for the following reasons: COVID-19 (n=4), early dismissal (n=12), psychiatric comorbidities not detected earlier (n=3), and treatment discontinuation due to a high incidence of COVID-19 cases at the trial center (n=12).

Of the 42 included patients, 17 (28%) had a right-sided pathology, 22 (52%) had left-sided pathology, and 3 (7%) had damage occurring in both brain hemispheres.

**Table 1:** Types of brain damage.

Brain region	Patients, n
Cerebral infarction	33
Brain hemorrhage	3
Subarachnoid hemorrhage	2
Basal ganglia hemorrhage	4

**Table 2:** Demographic characteristics of control and VR^a^ group.

Variable		Control group (n=21)	VR patients (n=21)	*P* value	
Age (years), mean (SD; range)		67.3 (4.6; 59-74)	68.3 (14.5; 36-93)	.69	
MMST^b^, mean (SD; range)		26.95 (2.04; 24-30)	27.05 (2.11; 24-30)	.97	
**Sex, n (%)**					.67
	Female	8 (38)	6 (29)		
	Male	13 (62)	15 (71)		

^a^VR: virtual reality.

^b^MMST: Mini Mental State Test.

**Table 3:** Mean performance and SD in the control and VRa group for all measures used in the trial at baseline.

Measure	Control group, mean (SD; range)	VR patients, mean (SD; range)
TMT^a^ A	93.7 (47.83; 36-205)	80.95 (47.84; 39-200)
TMT B	326.9 (216.7; 91-853)	29 2.2 (244.3; 96-1056)
TL-D^b^	54.9 (31; 3-99)	42.62 (32.1; 1-99)
EQ-5D VAS^c^	45.5 (21.02; 0-80)	49.05 (17.22; 0-75)
AKT_T_PR^d^	43.04 (21.4; 0-74.4)	41.07 (21.8; 0-97)
WMS-R^e^ forward	39.6 (29.23; 0-98)	41.9 (34.99; 0-98)
WMS-R backward	28.2 (29.82; 0-98)	36.7 (32.96; 2-97)
SF-36^f^ energy	42.83 (20.47; 0-80)	42.38 (20.77; 5-80)
SF-36 emotional well-being	55.15 (22.65; 0-88)	64.24 (23.94; 16-100)
SF-36 social functioning	36.87 (26.74; 0-100)	50.6 (31.99; 0-100)
SF-36 pain	51.75 (34.57; 0-100)	63.88 (34.6; 0-100)
SF-36 general health	47.5 (14.18; 25-75)	46.85 (13.82; 10-70)
SF-36 physical function	29.75 (30.45; 0-100)	20.77 (20.08; 0-66.6)
SF-36 role limitation in physical health	22.37 (36.22; 0-100)	13.49 (30.44; 0-100)
SF-36 role limitation emotional problems	35.09 (45.1; 0-100)	31.66 (43.9; 0-100)

^a^TMT: Trail Making Test.

^b^TL-D: Tower of London—German version.

^c^VAS: visual analog scale.

^d^AKT_T_PR: Alters-Konzentrations-Test time percentile rank.

^e^WMS-R: Wechsler Memory Scale—Revised.

^f^SF-36: Short Form 36.

### Nausea and Motion Sickness

Of the 21 patients in the VR group, none reported problems with motion sickness or associated nausea, and no treatment had to be stopped due to motion sickness or nausea. This is congruent with the results of our feasibility trial with patients with stroke and healthy older adults, where we demonstrated that applying specific principles in VR development, such as having complete control of all motions in VR, reduces the risk of experiencing motion sickness and is feasible for use in special needs groups such as patients with stroke [16]. Patients in the control group using CCT reported no negative side effects. The patients conducted, on average, 18.7 (SD 0.99) successful treatment sessions.

### Treatment Satisfaction

Patients were asked as part of the FEPVR tool to rate the treatment satisfaction on a Likert scale ranging from 1 (*very dissatisfied*) to 7 (*very satisfied*). Both groups reported a tendency toward high acceptance and satisfaction with both treatments (M=5.7). The treatment also created the impression that it helped against the present condition in both groups (VR: M=5.5, CCT: M=5.7), rated from 1 (*I completely disagree*) to 7 (*I fully agree*).

### Adverse Events

While the trial was conducted, no severe adverse event or adverse event was linked to the intervention in either group; nevertheless, we had to report adverse events as the clinical trial was conducted amid the COVID-19 pandemic in 2020 and 2021. Owing to the general incidence of infections in Germany, 4 patients included in the trial were infected with SARS-CoV-2 and were isolated and excluded from the study.

### Survey Completion

Of the patients who completed the study, 97% (41/42) of the participants completed all the questionnaires and tests, with only occasional missing items or tests. This might be explained by the fact that all surveys were conducted in the presence of a therapist or neuropsychologist working and trained in the clinic.

### Cognitive Tests

To evaluate the impact of both treatments on cognitive status, subjective health, and quality of life, we used *SPSS Statistics* (version 29, IBM) and calculated repeated-measures ANOVA. For the comparisons between the 2 groups between the pre- and posttest, repeated-measures ANOVA and Bonferroni post hoc test indicated that TL-D scores significantly differed in the VR group after the intervention (*P*=.046). Table 4 presents the results of all other conducted tests. As a nonsignificant trend emerged in the TMT A (main effect: *P*=.01; time by group interaction: *P*=.09) and WMS-R (main effect: *P*=.006; time by group interaction: *P*=.18) results, a post hoc test was also performed. No further post hoc tests were performed for the other variables.

**Table 4:** Clinical parameters of the virtual reality (VR) and computerized cognitive training groups before and after the intervention.

Measure and time point			Control group^a^ (n=21)		VR group^b^ (n=21)
**TMT^c^ A scores, mean (SD; range)^d^**					
	Pretreatment	93.7 (47.83; 36-205)		80.95 (47.84; 39-200)	
	Posttreatment	89.95 (55.2; 26-257)		61 (22.87; 32-135)	
**TMT B scores, mean (SD; range)^e^**					
	Pretreatment	326.9 (216.7; 91-853)		292.24 (244.26; 96-1056)	
	Posttreatment	283.75 (198.86; 64-877)		268.29 (217.6; 99-855)	
**WMS-R^f^, mean (SD; range)^g^**					
	Pretreatment	39.6 (29.23; 0-98)		41.9 (34.99; 0-98)	
	Posttreatment	44.75 (31.24; 0-88)		55.95 (30.29; 0-98)	
**TL-D^h^ scores, mean (SD; range)^i^**					
	Pretreatment	54.9 (31; 3-99)		42.62 (32.1; 1-99)	
	Posttreatment	52.2 (33.7; 2-99)		73.1 (32.5; 7-99)	
**EQ-5D VAS^j^ scores, mean (SD; range)^k^**					
	Pretreatment	45.5 (21.55; 0-80)		49.05 (17.22; 0-75)	
	Posttreatment	46.84 (25.01; 0-80)		62.86 (21.3; 10-95)	
**AKT^l^ scores, mean (SD; range)^m^**					
	Pretreatment	87.47 (62.98; 32-281)		89.23 (69.57; 28-357)	
	Posttreatment	93.15 (67.83; 33-351)		87.86 (69.56; 35-350)	
**SF-36^n^ energy domain, mean (SD; range)^o^**					
	Pretreatment	42.83 (20.47; 0-80)		42.38 (20.77; 5-80)	
	Posttreatment	51.32 (20.27; 15-90)		47.86 (21.3; 10-85)	
**SF-36 emotional well-being domain, mean (SD; range)**					
	Pretreatment	55.15 (22.65; 0-88)		64.24 (23.94; 16-100)	
	Posttreatment	64.63 (17.55; 32-100)		66.1 (22.36; 24-100)	
**SF-36 social functioning domain, mean (SD; range)**					
	Pretreatment	36.87 (26.74; 0-100)		50.6 (31.99; 0-100)	
	Posttreatment	48.13 (28.76; 0-100)		54.17 (34.08; 0-100)	
**SF-36 pain domain, mean (SD; range)**					
	Pretreatment	51.75 (34.57; 0-100)		63.88 (34.6; 0-100)	
	Posttreatment	59.38 (30.27; 20-100)		60.48 (33.3; 0-100)	
**SF-36 general health domain, mean (SD; range)**					
	Pretreatment	47.5 (14.2; 25-75)		46.85 (13.82; 10-70)	
	Posttreatment	48.25 (16.8; 10-90)		48.57 (16.52; 20-90)	
**SF-36 physical functioning domain, mean (SD; range)**					
	Pretreatment	29.75 (30.46; 0-100)		20.77 (20.07; 0-66.7)	
	Posttreatment	27.89 (21.99; 0-65)		36.43 (29.33; 0-95)	
**SF-36 role limitation physical health domain, mean (SD; range)**					
	Pretreatment	22.37 (36.22; 0-100)		13.49 (30.44; 0-100)	
	Posttreatment	21.25 (35.61; 0-100)		15 (31.83; 0-100)	
**SF-36 role limitation emotional problems, mean (SD; range)**					
	Pretreatment	35.09 (45.1; 0-100)		31.66 (43.9; 0-100)	
	Posttreatment	45.33 (49.68; 0-100)		42.86 (48.47; 0-100)	

^a^Control group using computerized cognitive training.

^b^VR group using immersive VR cognitive training.

^c^TMT: Trail Making Test.

^d^Repeated-measures ANOVA: no significant interaction detected (*P*=.09).

^e^Repeated-measures ANOVA: no significant interaction detected (*P*=.68).

^f^WMS-R: Wechsler Memory Scale—Revised.

^g^Repeated measures analysis of variance: no significant interaction detected (*P*=.18).

^h^TL-D: Tower of London.

^i^Repeated-measures ANOVA: time, *F*_1,39_=4.255; *P*=.046 (significant); time × group, *F*_1,39_=6.07; *P*=.02 (significant). A Bonferroni post hoc test was significant between the VR group at pretest and the VR group at posttest (*P_bonf_*=.02; η²=0.135).

^j^VAS: visual analog scale.

^k^Repeated-measures ANOVA: no significant interaction detected (*P*=.16).

^l^AKT: Alters-Konzentrations-Test.

^m^Repeated-measures ANOVA: no significant interaction detected (*P=*.52).

^n^SF-36: Short Form 36.

^o^Repeated-measures ANOVA: no significant interaction detected (*P_pain_*=.28; *P_energy_*=.69; *P_emo_wb_*=.31; *P_soc_func_*=.59; *P_gen_health_*=.87; *P_phys_func_*=.06; *P_role_lim_phys_*=.88; *P_role_lim_emo_*=.69).

### PANAS Trial

Patients were asked to describe their affects on the allocated treatment after 7 sessions of trial participation. Affect was measured with the German version of PANAS, and patients were asked to rate their affect immediately after treatment. An ANOVA was performed, resulting in *F*_1,36_=0.879 (*P*=.63) for the positive affect scale and *F*_1,36_=0.954 (*P*=.57) for the negative affect scale. The test results showed no difference between the intervention and control groups regarding positive and negative affect toward the kind of treatment received by both groups during the trial. Both groups reported high positive affect, with the VR group at mean 29.809 (SD 7.98) and the control group at mean 25.6 (SD 8.23), showing a slightly higher positive affect in favor of the VR group. Negative affect results were rated mean 15.95 (SD 8.59) in the VR group and mean 15.66 (SD 6.18) in the control group, showing similar negative affect ratings in both groups.

### Summary

**Figure 4:**
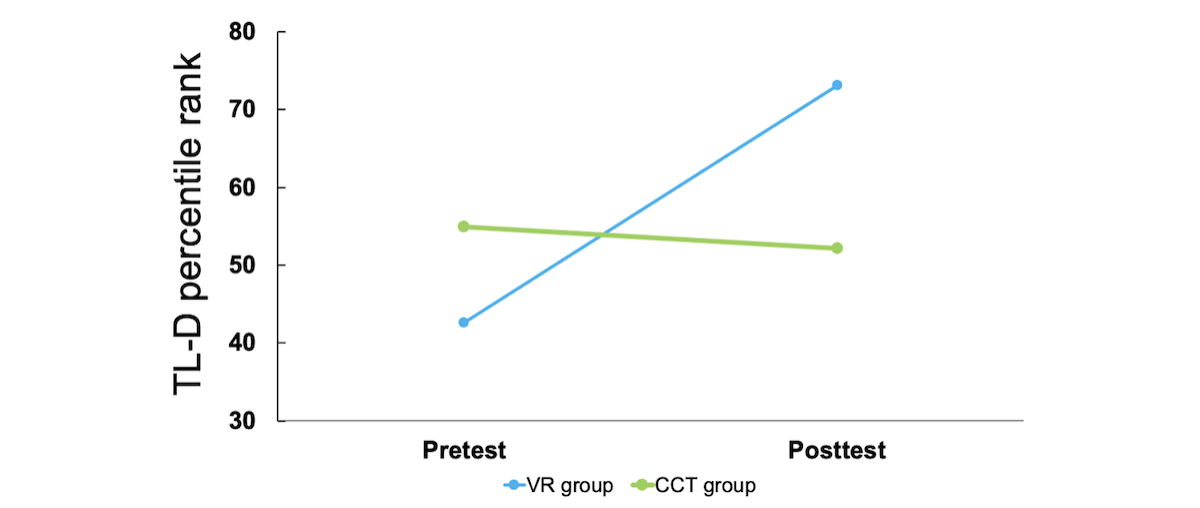
Results of repeated measures analysis of variance Tower of London—German version (TL-D). CCT: computerized cognitive training; VR: virtual reality.

The critical effects for both groups are presented in Table 4, showing mean changes between *t_0_* and *t_1_* and the results of repeated-measures ANOVA for group effects. Figure 4 shows the mean changes for TL-D results in both groups.

## Discussion

### Overview

To our knowledge, this is one of the few trials comparing immersive VR cognitive training, incorporating ADL and SG and focusing on the ability to perform ADL, with a multitude of cognitive domains and not single domains only, with CCT. In this randomized controlled trial, we aimed to determine the impact of both cognitive training in immersive VR and CCT on cognition and the ability to perform ADL in patients in inpatient neurological rehabilitation with an MMST score >20 in rehabilitation phases C and D, combining immersive VR with paradigms of neuropsychology and occupational therapy. In the patient group receiving VR therapy, we found a statistically significant improvement in executive functioning within the 4-week treatment interval. Some nonsignificant trends were detected for attention and processing speed, working memory, and subjective health. No significant changes were observed in the control group.

In recent years, VR systems found their way to neurological rehabilitation. However, being used most often for motor rehabilitation, only a few systems have been developed and evaluated concerning their impact on cognition in patients with acute brain affection. Furthermore, some developed trainings have only been used for research purposes but have yet to undergo more extensive testing or application in clinical practice. In addition, several cognitive trainings in VR were developed to target a specific cognitive domain, for example, memory [38] and attention [39].

### Principal Findings

We compared the VR group with a control group using CCT, which represents care as usual in German rehabilitation facilities. Both groups were able to engage in their assigned interventions successfully. Despite our initial concerns regarding the number of sessions to be conducted in VR, no significant side effects were reported. All patients participated in all sessions required to complete the trial. Some initial hurdles had to be overcome, for example, mainly introducing older adult patients to an entirely new technology or ensuring continuous disinfection of all equipment, as the trial took place at a time when the COVID-19 pandemic situation in Germany was very tense. However, after the first treatment session, those problems were solved without further external intervention. Overall, the acceptance and usability of both technologies were observably high, which, in terms of the VR experience, is congruent with the results of our feasibility study using an earlier version of the software used in this trial [16].

The demographics between the 2 groups were similar: not significantly different in age and pathology but with a larger proportion of male participants in the trial. The trial’s sample size was too small to make far-reaching statements, but clear tendencies provided the first indication of training effects that opened up room for further research in a larger trial.

We detected the effects of immersive VR on different cognitive functions in patients with acute brain affection receiving treatment during inpatient neurological rehabilitation. Comparing both groups by calculating repeated-measures ANOVA, we detected improvements in some cognitive domains in the VR group but no significant improvement in any cognitive function in the control group. By conducting the TL-D, specifically assessing problem-solving and planning, we found significant improvements from pre- to postintervention phase in the VR group, and the Bonferroni post hoc test also verified these significant improvements with a medium-sized effect of η^2^=0.135. Oliveira et al [40] conducted a trial to investigate the impact of VR on ADL in stroke rehabilitation. They found that VR training resulted in better tests measuring EF, global cognition, attention, and memory training. This partly overlaps with the results of this study. We also observed improvements in attention, processing speed, and subjective health status in the VR group. However, these did not become significant in the repeated-measures ANOVA, while the scores for the named variables persisted at the *t_0_*-level after the treatment in the control group. No significant improvements and differences between groups were detected in concentration and memory in both groups.

The improvements in subjective health state are of interest for further research, as Domínguez-Tellez et al [41] ascertained in their systematic review that VR interventions for upper limb rehabilitation were a part of improving quality of life. They hypothesized that an improvement in the actual pathology leads to better abilities for carrying out ADL, and an improvement in quality of life goes hand in hand with a higher grade of participation. The higher levels of subjective health state in the VR group (mean 62.86, SD 21.03) compared with the control group (mean 46.84, SD 25.01) after the intervention, with an improvement on the EQ-5D VAS-scale of 13.81 points in the VR group and 1.34 points in the control group between *t_0_* and *t_1_*, although not significantly different, showed a tendency toward the impact of VR treatment when considering the significant improvement in TL-D scores in the VR group and the resulting implications for participation in daily life. More thorough research is necessary to better understand the interrelationship between these constructs and the results of the SF-36 subscales.

Looking at the results the VR group achieved in planning and problem-solving (TL-D), we would have expected to notice a significant difference in TMT results as well, as both tests are positively correlated [42]. TMT specifically measures cognitive flexibility, alternating attention, sequencing, visual search, and motor speed, which overlap with the measured domains of TL-D, predominantly measuring planning and problem-solving. The missing significant improvement in the abilities measured by TMT in this trial raises the question of how specifically the VR training stimulates particular cognitive skills. We still see a tendency for improvement in these cognitive abilities, as the VR group achieved more improvement between *t_0_* and *t_1_* than the control group, as measured by TMT A. We found a nonsignificant trend in the repeated-measures ANOVA (*P*=.09). As we expected a correlation between TL-D and TMT results, we calculated a Bonferroni post hoc test, which resulted in a significant improvement in the VR group (Bonferroni *P*=.02). This is only a trend, which cannot be used to draw far-reaching conclusions. Nevertheless, it is interesting, as this might correlate with the significant improvement in TL-D measures in the same group. Nevertheless, it would be interesting to investigate in a larger trial if an optimized version of the training software leads to more diversified and measurable effects.

In addition, other studies cited in this paper have reported further improvements in multiple cognitive subdomains. In our study, significant improvements were found only in the EF. One explanation for the difference in results is that both groups received only 1 therapy to treat cognitive dysfunction rather than simply augmenting the existing treatment. A 2017 Cochrane Review found that in many studies, VR therapy was added to traditional treatment, resulting in a higher total amount of therapy [43]. Therefore, it cannot be said with certainty whether VR therapy alone is causal for the improvement in these studies or whether the overall increased measure of treatment compared with the control group is the determining factor. The fact that each group in our study received only 1 cognitive rehabilitation treatment means that the effects can be more precisely attributed to a single treatment method.

When developing the software used in collaboration with occupational therapists, neuropsychologists, neurologists, and patients, we were particularly faced with the lack of proximity to daily life of current CCT, which led us to the question of how we could depict everyday life and its challenges in a way that is easy to understand, therapeutically beneficial, and motivating, so that patients could adhere to training over a more extended period. The results showed the VR training used in this trial. The following components have played a significant role in its development and could provide a directional indicator for further development of new training methods for cognitive rehabilitation.

The results of this trial helped us formulate hypotheses on why VR-based training might lead to better results in cognitive rehabilitation than PC-based interventions:

### Ecological Validity

In rehabilitation, particularly when focusing on returning to daily life, it is vital to have ecologically valid training simulations, for which VR is well suited [40,44]. In our application, we presented a garden and kitchen scenario to simulate activities and situations in which subjects found themselves after rehabilitation. In reality, as it is either dangerous or unavailable, it is often not viable to let patients train in real-life settings. VR enables stimulation with settings as close as possible to reality, potentially facilitating a more effortless transfer from training to reality.

The results of this trial indicate that high ecological validity is beneficial for training cognition. Patients in the VR group achieved significantly higher scores in planning and problem-solving than their counterparts in the control group. In addition, they reported better scores in terms of speed and health status than the conventionally treated patients. This is congruent with the results of Faria et al [21], who examined the benefits of immersive ADL simulation in neurological rehabilitation [21].

### Gamification and Immersion

Gamification adds games or game-like elements to encourage participation or engagement. Engagement is a crucial component of rehabilitation; in patients with stroke, it increases the rate of rehabilitation and rehabilitation outcomes of survivors of stroke [45]. The VR experience relies to a great extent on the grade of immersion. Immersion describes the effect of a virtual environment that causes the user’s awareness of being exposed to illusory stimuli to fade into the background to such an extent that the virtual world is perceived as real. High-grade immersion can be created using HMD-VR. The user wears device-like goggles on the head over the eyes, effectively masking the actual environment.

Combining these 2 aspects and adding up the feature of receiving immediate feedback from the virtual environment on performance leads to increased motivation and, subsequently, patients stuck with the treatment longer [43]. Yoshida et al [46] discussed motivation in patients with subacute stroke and discovered that it is mainly influenced by extrinsic reward factors (eg, positive feedback provided by therapists). However, if basic psychological needs are met, for example, autonomy or competence, which play a critical role in stroke rehabilitation, the motivation pattern switches from extrinsic to intrinsic [46]. Long after an acute event, improvements in rehabilitation can still be achieved, and patients’ self-perception regarding the improvements is paramount.

### On the Right Dosage

Participants in this trial received a treatment dosage of 18.7 sessions over 4 to 6 weeks, each lasting 30 minutes (9.35 hours per participant). Other trials reported an average intervention duration of 13.3 hours [39,40,47-50], ranging from 4 to 24 hours, with a session duration of 30 to 60 minutes, 3 to 5 times a week. Our dosing was sufficient to achieve a measurable effect in some domains and was also oriented toward the care reality in German rehabilitation hospitals. To attain more significant outcomes in multiple domains, it would be interesting to see if other training tasks in immersive VR create an effect on cognition and if a varying number of treatment sessions beyond inpatient care leads to different results than those reported here. On the basis of the results of this trial, we conclude that 18 to 19 sessions over 30 minutes are sufficient to achieve a measurable treatment effect. Still, we cannot determine whether fewer sessions would create similar effects if more treatment automatically leads to more significant outcomes and if those effects vary between patient groups. More extensive trials with blinding, subgroups, and longer treatment duration are necessary to answer this question.

### Transfer to Daily Life and How to Measure it

One end point of this trial, which we were not able to meet, was to identify whether the treatments in both groups induced transfer effects on daily life by examining if patients in either group were in a better state to perform instrumental ADL, such as preparing food or handling the washing machine, after the intervention. While designing this trial, we noted that standardized neuropsychological testing batteries are well suited to test particular cognitive domains and performance therein, but that daily life is more complex—it can not be reduced to 1 or 2 cognitive processes at the same time—and that functional capabilities of managing everyday life as the end goal of rehabilitation are not sufficiently represented by those testing batteries when focusing on cognitive rehabilitation [40]. Therefore, we developed a testing tool, FEPVR. It was used for the first time in this trial, and we faced some issues with properly capturing the ADL. We plan to publish the battery and its manual in a revised version, including experiences from use in this trial so that further development and testing by other research groups is possible.

### Limitations

Although this study provides valuable insights into the impact of immersive VR on cognitive rehabilitation, several questions remain unanswered. Specifically, we identified the following limitations that should be considered: future research in this field should be conducted with larger sample groups. This randomized controlled pilot study builds a profound understanding of the potential immersive VR can have in neurological rehabilitation for cognitive impairment in patients with MMST scores >20, affected by stroke, to the largest extent. Conclusions cannot be drawn for patients below this cut-off score. We also need more information on the manifestation of training effects, for example, if the measured results are still the same 6 to 12 months after the intervention and if an actual transfer effect to ADL occurs. Therefore, long-term studies with follow-up over multiple years are necessary.

The applied VR environment was limited to only 2 training scenarios inspired by ADL. More training scenarios replicating other ADL should be developed and tested in future studies.

Even though there are findings about the application of VR in rehabilitation of other patient groups, for example, multiple sclerosis, psychiatric disorders, and pain management [51], the application used here has chiefly been tested in the rehabilitation of patients with acute brain affection. Therefore, the evaluation needs to consider patients from the abovementioned groups to draw further conclusions in other patient groups.

### Conclusions and Outlook

Our group of psychologists, software developers, 3D artists, neurologists, and occupational therapists has developed a new form of immersive VR-based cognitive rehabilitation training. After proving the feasibility and acceptability in stroke patients with stroke, we demonstrated the initial therapeutic effects of a novel immersive VR training on EF in patients undergoing inpatient neurological rehabilitation. Data were collected over 18 months, including multiple recruitment pauses due to the COVID-19 pandemic between 2020 and 2022. The supervision of patients was necessary for conducting treatment, but it was administered by trained therapy assistants, freeing time for neuropsychologists and occupational therapists. None of the patients in the VR group had previously been exposed to VR, but that did not diminish their excitement about receiving the experimental treatment and getting the most out of it.

It was reported earlier that combining cognitive training with physical activity leads to even better results; for example, a group of older adults with mild cognitive impairment showed more significant improvements in multiple cognitive domains when receiving combined therapy than physical or cognitive training alone [52]. Investigating whether the treatment effects of VR-based cognitive training can be increased by additional full-body physical training would be an interesting research topic for future trials. We also intend to depict everyday life in VR even more with new training scenarios, a higher level of gamification, and using techniques to personalize the treatment itself to a greater extent.

Our goal is to ensure that treatments in neurorehabilitation are improved and that all parties involved profit from new developments. The excellent acceptance, low side effects, and initial therapeutic effects of immersive VR treatment presented here offer new therapeutic options, for example, applying SG in routine therapy [53]. The results of this pilot trial show that a short but intense period of treatment leads to measurable improvement in EF, a manifold construct consisting of behavioral and higher-order cognitive skills necessary for independent functioning and carrying out everyday life, such as shopping, managing money, and preparing meals [3,8]. It would be interesting to determine if these results can be extended if patients continue treatment at home and increase the overall number of treatment sessions.

Temehy et al [4] explored the needs of patients with stroke after discharge from rehabilitation; they discovered that patients often not only need physical rehabilitation but also psychological services, comprising cognitive rehabilitation. Shipley et al [54] cite a patient who says, “it should have been a formal process to gain access to a psychologist, and it would have been beneficial from an earlier time point, such as from the acute hospital.” Access to qualified and trained neuropsychologists in Germany is difficult, as the *Gemeinsamer Bundesausschuss* (Federal Joint Committee) states that only 1100 neuropsychologists in outpatient care are available. The 2019 German Society for Neuropsychology list contains even fewer specialists with only 350 practitioners in outpatient settings [55]. This shortcoming highlights the need for complementary treatment approaches. This is in line with what the COVID-19 pandemic has uncovered: the need for digitized rehabilitation tools, bringing the general problem of accessibility of rehabilitation services to center, for example, for people living in rural areas or simply an insufficient number of neuropsychologists or occupational therapists in Germany, which prevents the actual implementation of necessary aftercare in neurological rehabilitation with a regular high number of treatment sessions for manifesting rehabilitation progress. Further development of video game–based rehabilitation approaches with digitized platforms to bring care home should be the focus of other groups active in this field, which is also emphasized by Ong et al [56] and Salisbury et al [57], who stated that VR is a promising technology to foster home care and shorten hospitalization. As prices for HMD-VR devices continuously decrease and the acceptance of digitized treatments in Germany increases, it augments the chance that access to VR-assisted rehabilitation will become more accessible and a standard in a wide range of treatment settings.

## Appendix

Multimedia Appendix 1 Greenhouse screenshot 1.

Multimedia Appendix 2 Greenhouse screenshot 2.

Multimedia Appendix 3 Kitchen 1.

Multimedia Appendix 4 Small kitchen lobby.

Multimedia Appendix 5 CONSORT-EHEALTH checklist (V. 1.6.1).

## Abbreviations

ADL: activities of daily living
AKT: Alters-Konzentrations-Test
CCT: computerized cognitive training
CONSORT: Consolidated Standards of Reporting Trials
EF: executive functions
FEPVR: Fragebogen zur Erfassung der Performance in VR
HMD: head-mounted display
MMST: Mini Mental Status Test
PANAS: Positive and Negative Affect Schedule
SF-36: Short Form 36
SG: serious game
TL-D: Tower of London—German version
TMT: Trail Making Test
VAS: visual analog scale
VR: virtual reality
WMS-R: Wechsler Memory Scale—Revised

## Data Availability

The data sets generated during this study are available from the corresponding author only upon reasonable request.
