# Supplementary figures and images for: Cognitive Training With Head-Mounted Display Virtual Reality in Neurorehabilitation: Pilot Randomized Controlled Trial
Source: JMIR Serious Games. 2023 Jul 21;11:e45816. doi: 10.2196/45816 (PMC10403796; doi:10.2196/45816)

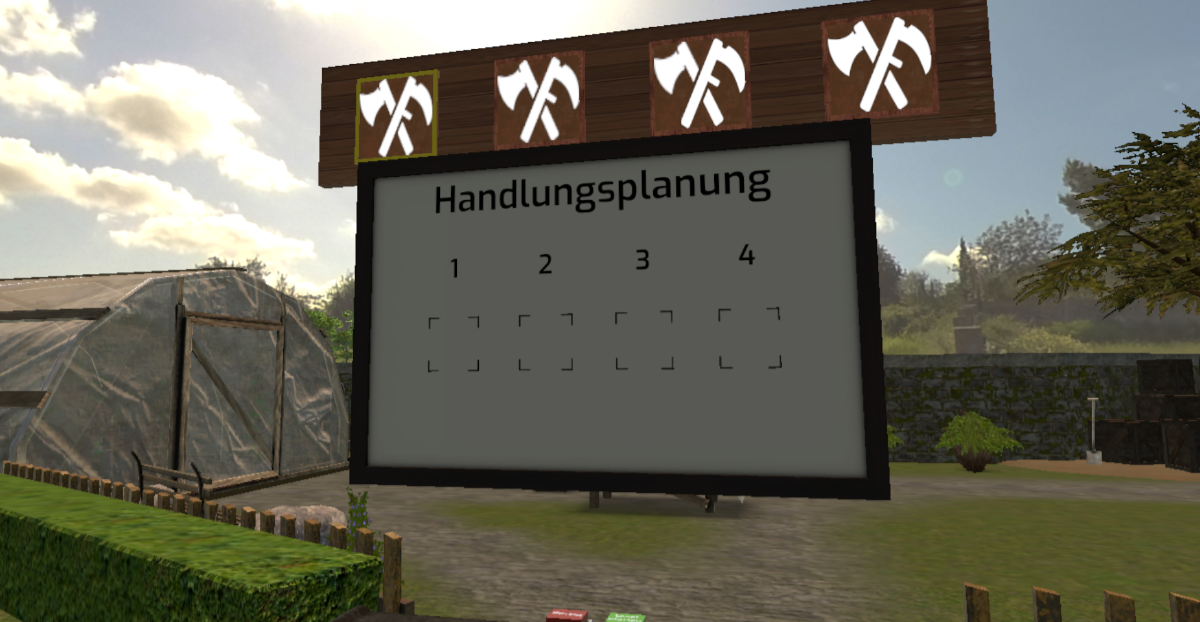

Supplement: Multimedia Appendix 1 [file games_v11i1e45816_app1.png]

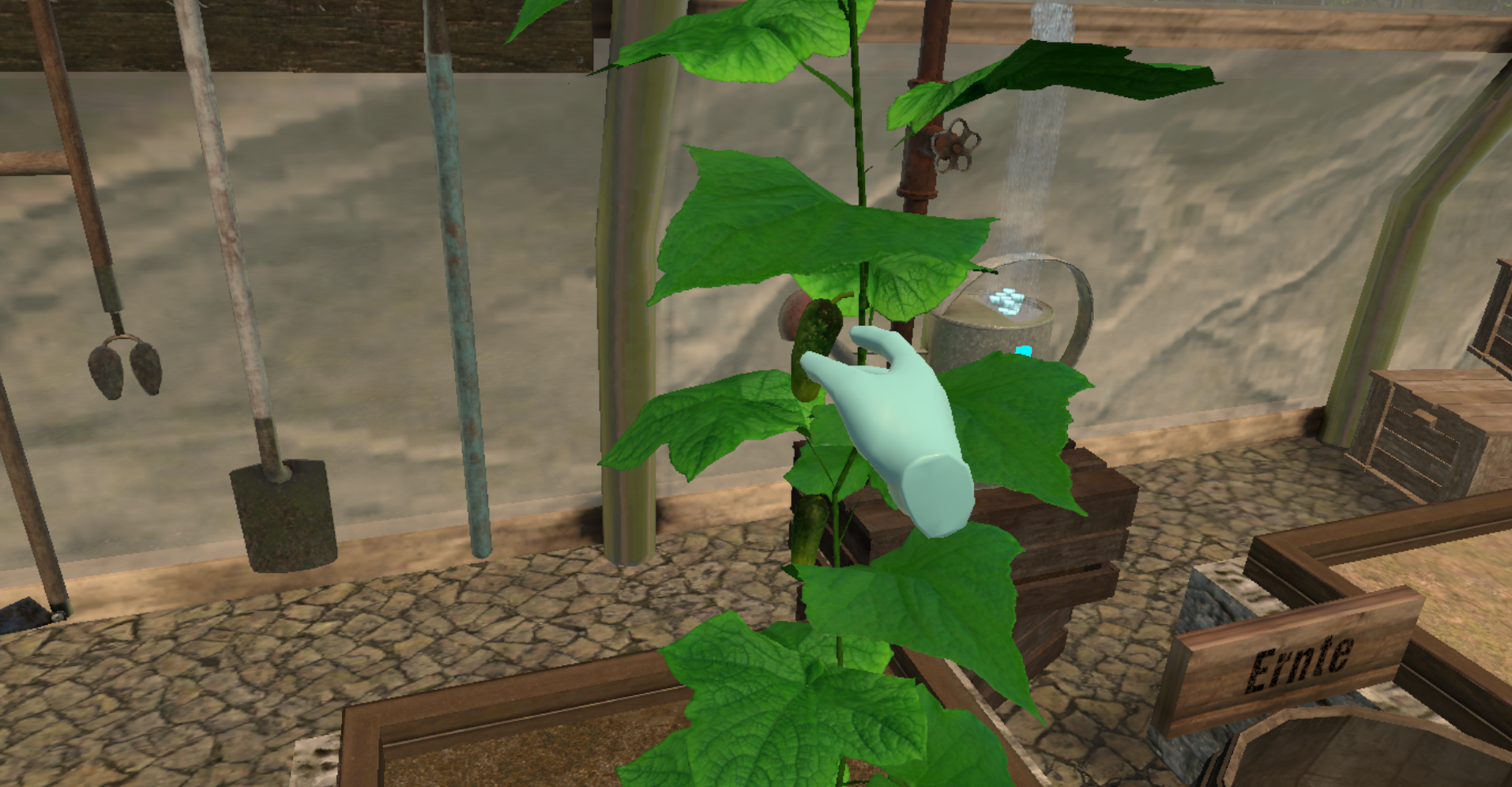

Supplement: Multimedia Appendix 2 [file games_v11i1e45816_app2.png]

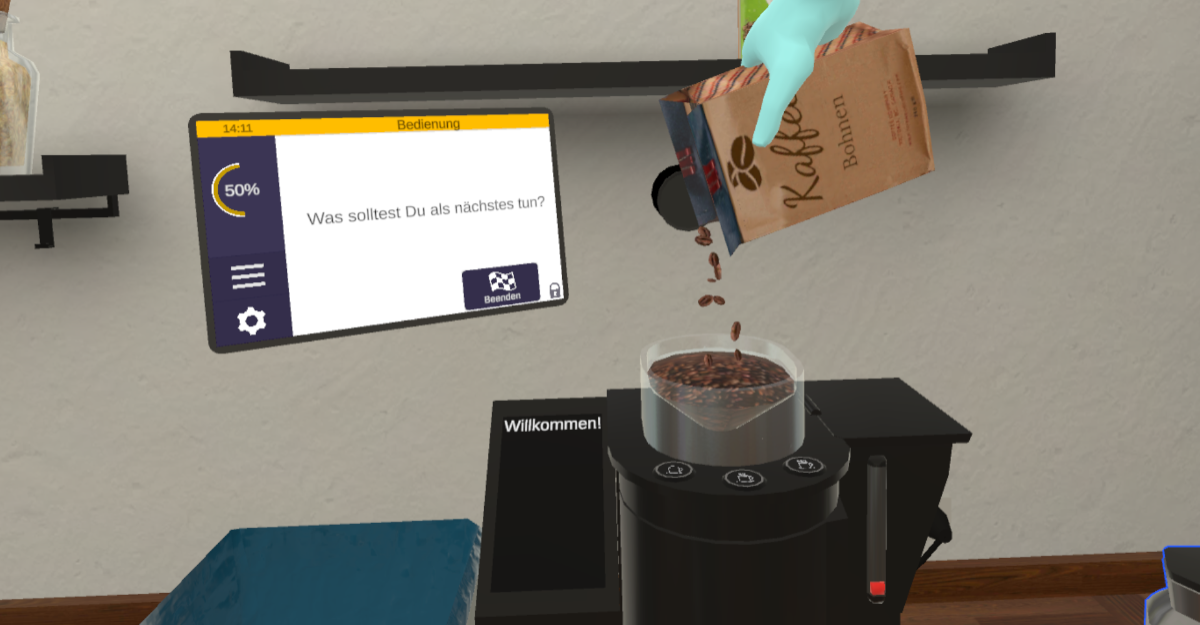

Supplement: Multimedia Appendix 3 [file games_v11i1e45816_app3.png]

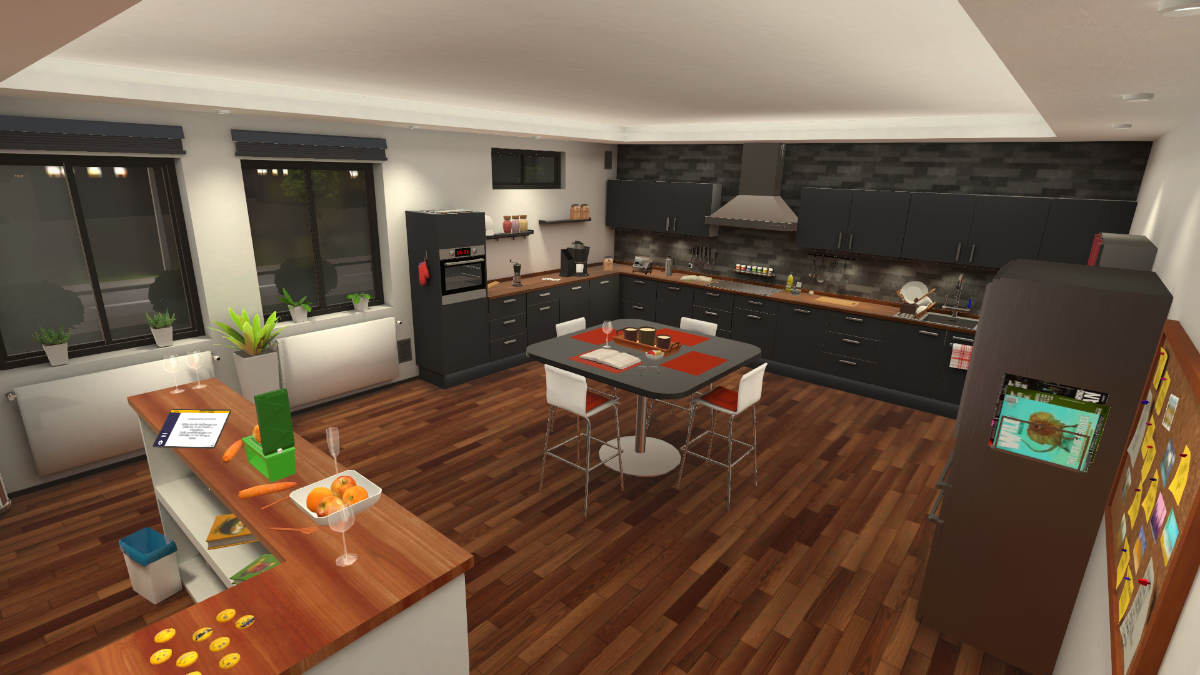

Supplement: Multimedia Appendix 4 [file games_v11i1e45816_app4.png]
