# Supplementary material for: Cognitive Training With Head-Mounted Display Virtual Reality in Neurorehabilitation: Pilot Randomized Controlled Trial
Source: JMIR Serious Games. 2023 Jul 21;11:e45816. doi: 10.2196/45816 (PMC10403796; doi:10.2196/45816)
Supplement: Multimedia Appendix 5 [file games_v11i1e45816_app5.pdf]

# CONSORT-EHEALTH (V 1.6.1) - Submission/Publication Form

The CONSORT-EHEALTH checklist is intended for authors of randomized trials evaluating web-based and Internet-based applications/interventions, including mobile interventions, electronic games (incl multiplayer games), social media, certain telehealth applications, and other interactive and/or networked electronic applications. Some of the items (e.g. all subitems under item 5 - description of the intervention) may also be applicable for other study designs.

The goal of the CONSORT EHEALTH checklist and guideline is to be
a) a guide for reporting for authors of RCTs,
b) to form a basis for appraisal of an ehealth trial (in terms of validity)

CONSORT-EHEALTH items/subitems are MANDATORY reporting items for studies published in the Journal of Medical Internet Research and other journals / scientific societies endorsing the checklist.

Items numbered 1., 2., 3., 4a., 4b etc are original CONSORT or CONSORT-NPT (non-pharmacologic treatment) items.
Items with Roman numerals (i., ii, iii, iv etc.) are CONSORT-EHEALTH extensions/clarifications.

As the CONSORT-EHEALTH checklist is still considered in a formative stage, we would ask that you also RATE ON A SCALE OF 1-5 how important/useful you feel each item is FOR THE PURPOSE OF THE CHECKLIST and reporting guideline (optional).

Mandatory reporting items are marked with a red *.
In the textboxes, either copy & paste the relevant sections from your manuscript into this form - please include any quotes from your manuscript in QUOTATION MARKS,
or answer directly by providing additional information not in the manuscript, or elaborating on why the item was not relevant for this study.

YOUR ANSWERS WILL BE PUBLISHED AS A SUPPLEMENTARY FILE TO YOUR PUBLICATION IN JMIR AND ARE CONSIDERED PART OF YOUR PUBLICATION (IF ACCEPTED).
Please fill in these questions diligently. Information will not be copyedited, so please use proper spelling and grammar, use correct capitalization, and avoid abbreviations.

DO NOT FORGET TO SAVE AS PDF _AND_ CLICK THE SUBMIT BUTTON SO YOUR ANSWERS ARE IN OUR DATABASE !!!

Citation Suggestion (if you append the pdf as Appendix we suggest to cite this paper in the caption):
Eysenbach G, CONSORT-EHEALTH Group
CONSORT-EHEALTH: Improving and Standardizing Evaluation Reports of Web-based and Mobile Health Interventions
J Med Internet Res 2011;13(4):e126

URL: http://www.jmir.org/2011/4/e126/
doi: 10.2196/jmir.1923
PMID: 22209829

julian.specht@livingbrain.de Konto wechseln

Nicht freigegeben

Entwurf gespeichert

* Gibt eine erforderliche Frage an

Your name *

First Last

Julian Specht

Primary Affiliation (short), City, Country *

University of Toronto, Toronto, Canada

SRH Hochschule Heidelberg, Heidelberg, Germ

Your e-mail address *

abc@gmail.com

julian.specht@livingbrain.de

Title of your manuscript *

Provide the (draft) title of your manuscript.

Head-mounted display virtual reality cognitive training in neurorehabilitation: Randomized controlled pilot study

Name of your App/Software/Intervention *

If there is a short and a long/alternate name, write the short name first and add the long name in brackets.

teora mind

Evaluated Version (if any)

e.g. "V1", "Release 2017-03-01", "Version 2.0.27913"

Meine Antwort

Language(s) *

What language is the intervention/app in? If multiple languages are available, separate by comma (e.g. "English, French")

German

URL of your Intervention Website or App

e.g. a direct link to the mobile app on app in appstore (itunes, Google Play), or URL of the website. If the intervention is a DVD or hardware, you can also link to an Amazon page.

Meine Antwort

URL of an image/screenshot (optional)

Meine Antwort

### Accessibility *

Can an enduser access the intervention presently?

- ○ access is free and open
- ○ access only for special usergroups, not open
- ◉ access is open to everyone, but requires payment/subscription/in-app purchases
- ○ app/intervention no longer accessible
- ○ Sonstiges:

### Primary Medical Indication/Disease/Condition *

e.g. "Stress", "Diabetes", or define the target group in brackets after the condition, e.g. "Autism (Parents of children with)", "Alzheimers (Informal Caregivers of)"

cognitive impairment in brain disease

### Primary Outcomes measured in trial *

comma-separated list of primary outcomes reported in the trial

impact of an ADL-based VR-SG on patients' co

### Secondary/other outcomes

Are there any other outcomes the intervention is expected to affect?

impact of an ADL-based VR serious game on self-reported outcomes, quality of life, state of health, affect in comparison to a conventional computerized cognitive training treatment.

Recommended "Dose" *

What do the instructions for users say on how often the app should be used?

- (x) Approximately Daily
- ( ) Approximately Weekly
- ( ) Approximately Monthly
- ( ) Approximately Yearly
- ( ) "as needed"
- ( ) Sonstiges:

Approx. Percentage of Users (starters) still using the app as recommended after 3 months *

- ( ) unknown / not evaluated
- ( ) 0-10%
- ( ) 11-20%
- ( ) 21-30%
- ( ) 31-40%
- ( ) 41-50%
- ( ) 51-60%
- ( ) 61-70%
- ( ) 71%-80%
- ( ) 81-90%
- ( ) 91-100%
- (x) Sonstiges: not applicable, as the use was for a specific user group in inpatier

Overall, was the app/intervention effective? *

- ( ) yes: all primary outcomes were significantly better in intervention group vs control
- (•) partly: SOME primary outcomes were significantly better in intervention group vs control
- ( ) no statistically significant difference between control and intervention
- ( ) potentially harmful: control was significantly better than intervention in one or more outcomes
- ( ) inconclusive: more research is needed
- ( ) Sonstiges:

Article Preparation Status/Stage *

At which stage in your article preparation are you currently (at the time you fill in this form)

- ( ) not submitted yet - in early draft status
- ( ) not submitted yet - in late draft status, just before submission
- ( ) submitted to a journal but not reviewed yet
- ( ) submitted to a journal and after receiving initial reviewer comments
- (•) submitted to a journal and accepted, but not published yet
- ( ) published
- ( ) Sonstiges:

Journal *

If you already know where you will submit this paper (or if it is already submitted), please provide the journal name (if it is not JMIR, provide the journal name under "other")

- ○ not submitted yet / unclear where I will submit this
- ○ Journal of Medical Internet Research (JMIR)
- ○ JMIR mHealth and UHealth
- ◉ JMIR Serious Games
- ○ JMIR Mental Health
- ○ JMIR Public Health
- ○ JMIR Formative Research
- ○ Other JMIR sister journal
- ○ Sonstiges:

Is this a full powered effectiveness trial or a pilot/feasibility trial? *

- ◉ Pilot/feasibility
- ○ Fully powered

Manuscript tracking number *

If this is a JMIR submission, please provide the manuscript tracking number under "other" (The ms tracking number can be found in the submission acknowledgement email, or when you login as author in JMIR. If the paper is already published in JMIR, then the ms tracking number is the four-digit number at the end of the DOI, to be found at the bottom of each published article in JMIR)

- ○ no ms number (yet) / not (yet) submitted to / published in JMIR
- ◉ Sonstiges: 45816

## TITLE AND ABSTRACT

### 1a) TITLE: Identification as a randomized trial in the title

1a) Does your paper address CONSORT item 1a? *

I.e does the title contain the phrase "Randomized Controlled Trial"? (if not, explain the reason under "other")

- (x) yes
- ( ) Sonstiges:

1a-i) Identify the mode of delivery in the title

Identify the mode of delivery. Preferably use “web-based” and/or “mobile” and/or “electronic game” in the title. Avoid ambiguous terms like “online”, “virtual”, “interactive”. Use “Internet-based” only if Intervention includes non-web-based Internet components (e.g. email), use “computer-based” or “electronic” only if offline products are used. Use “virtual” only in the context of “virtual reality” (3-D worlds). Use “online” only in the context of “online support groups”. Complement or substitute product names with broader terms for the class of products (such as “mobile” or “smart phone” instead of “iphone”), especially if the application runs on different platforms.

subitem not at all important

- 1 ( )
- 2 ( )
- 3 ( )
- 4 ( )
- 5 (x)

essential

Auswahl löschen

Does your paper address subitem 1a-i? *

Copy and paste relevant sections from manuscript title (include quotes in quotation marks "like this" to indicate direct quotes from your manuscript), or elaborate on this item by providing additional information not in the ms, or briefly explain why the item is not applicable/relevant for your study

Head-mounted display "virtual reality" cognitive training in neurorehabilitation: Randomized controlled pilot study

1a-ii) Non-web-based components or important co-interventions in title

Mention non-web-based components or important co-interventions in title, if any (e.g., “with telephone support”).

subitem not at all important

1 (selected)

2

3

4

5

essential

Auswahl löschen

Does your paper address subitem 1a-ii?

Copy and paste relevant sections from manuscript title (include quotes in quotation marks "like this" to indicate direct quotes from your manuscript), or elaborate on this item by providing additional information not in the ms, or briefly explain why the item is not applicable/relevant for your study

Meine Antwort

### 1a-iii) Primary condition or target group in the title

Mention primary condition or target group in the title, if any (e.g., “for children with Type I Diabetes”) Example: A Web-based and Mobile Intervention with Telephone Support for Children with Type I Diabetes: Randomized Controlled Trial

subitem not at all important

1 ○

2 ○

3 ○

4 ○

5 ◉

essential

Auswahl löschen

### Does your paper address subitem 1a-iii? *

Copy and paste relevant sections from manuscript title (include quotes in quotation marks "like this" to indicate direct quotes from your manuscript), or elaborate on this item by providing additional information not in the ms, or briefly explain why the item is not applicable/relevant for your study

Head-mounted display virtual reality cognitive training in "neurorehabilitation": Randomized controlled pilot study

### 1b) ABSTRACT: Structured summary of trial design, methods, results, and conclusions

NPT extension: Description of experimental treatment, comparator, care providers, centers, and blinding status.

1b-i) Key features/functionalities/components of the intervention and comparator in the METHODS section of the ABSTRACT

Mention key features/functionalities/components of the intervention and comparator in the abstract. If possible, also mention theories and principles used for designing the site. Keep in mind the needs of systematic reviewers and indexers by including important synonyms. (Note: Only report in the abstract what the main paper is reporting. If this information is missing from the main body of text, consider adding it)

subitem not at all important

1 ○

2 ○

3 ○

4 ○

5 ◉

essential

Auswahl löschen

Does your paper address subitem 1b-i? *

Copy and paste relevant sections from the manuscript abstract (include quotes in quotation marks "like this" to indicate direct quotes from your manuscript), or elaborate on this item by providing additional information not in the ms, or briefly explain why the item is not applicable/relevant for your study

"Participants were randomly assigned to two groups, one receiving the experimental VR treatment (N=21). The VR training consists of daily life scenarios, e.g., a kitchen, focusing on treating executive functions like planning and problem-solving. The control group (N=21) received conventional computerized cognitive training."

1b-ii) Level of human involvement in the METHODS section of the ABSTRACT

Clarify the level of human involvement in the abstract, e.g., use phrases like “fully automated” vs. “therapist/nurse/care provider/physician-assisted” (mention number and expertise of providers involved, if any). (Note: Only report in the abstract what the main paper is reporting. If this information is missing from the main body of text, consider adding it)

subitem not at all important

1 ○

2 ◉

3 ○

4 ○

5 ○

essential

Auswahl löschen

Does your paper address subitem 1b-ii?

Copy and paste relevant sections from the manuscript abstract (include quotes in quotation marks "like this" to indicate direct quotes from your manuscript), or elaborate on this item by providing additional information not in the ms, or briefly explain why the item is not applicable/relevant for your study

Meine Antwort

## 1b-iii) Open vs. closed, web-based (self-assessment) vs. face-to-face assessments in the METHODS section of the ABSTRACT

Mention how participants were recruited (online vs. offline), e.g., from an open access website or from a clinic or a closed online user group (closed usergroup trial), and clarify if this was a purely web-based trial, or there were face-to-face components (as part of the intervention or for assessment). Clearly say if outcomes were self-assessed through questionnaires (as common in web-based trials). Note: In traditional offline trials, an open trial (open-label trial) is a type of clinical trial in which both the researchers and participants know which treatment is being administered. To avoid confusion, use “blinded” or “unblinded” to indicated the level of blinding instead of “open”, as “open” in web-based trials usually refers to “open access” (i.e. participants can self-enrol). (Note: Only report in the abstract what the main paper is reporting. If this information is missing from the main body of text, consider adding it)

subitem not at all important

1 ○

2 ○

3 ○

4 ◉

5 ○

essential

Auswahl löschen

## Does your paper address subitem 1b-iii?

Copy and paste relevant sections from the manuscript abstract (include quotes in quotation marks "like this" to indicate direct quotes from your manuscript), or elaborate on this item by providing additional information not in the ms, or briefly explain why the item is not applicable/relevant for your study

"We recruited 42 patients with acute brain affection from a German neurorehabilitation clinic in inpatient care with MMST>20 to participate in this RCT."

1b-iv) RESULTS section in abstract must contain use data

Report number of participants enrolled/assessed in each group, the use/uptake of the intervention (e.g., attrition/adherence metrics, use over time, number of logins etc.), in addition to primary/secondary outcomes. (Note: Only report in the abstract what the main paper is reporting. If this information is missing from the main body of text, consider adding it)

subitem not at all important

1 ○

2 ○

3 ○

4 ○

5 ◉

essential

Auswahl löschen

Does your paper address subitem 1b-iv?

Copy and paste relevant sections from the manuscript abstract (include quotes in quotation marks "like this" to indicate direct quotes from your manuscript), or elaborate on this item by providing additional information not in the ms, or briefly explain why the item is not applicable/relevant for your study

"...42 patients with acute brain affection..." "The control group (N=21) received conventional computerized cognitive training."

1b-v) CONCLUSIONS/DISCUSSION in abstract for negative trials

Conclusions/Discussions in abstract for negative trials: Discuss the primary outcome - if the trial is negative (primary outcome not changed), and the intervention was not used, discuss whether negative results are attributable to lack of uptake and discuss reasons. (Note: Only report in the abstract what the main paper is reporting. If this information is missing from the main body of text, consider adding it)

subitem not at all important

1 ○

2 ○

3 ○

4 ○

5 ◉

essential

Auswahl löschen

Does your paper address subitem 1b-v?

Copy and paste relevant sections from the manuscript abstract (include quotes in quotation marks "like this" to indicate direct quotes from your manuscript), or elaborate on this item by providing additional information not in the ms, or briefly explain why the item is not applicable/relevant for your study

"The results of this trial indicate greater effectiveness for immersive VR training in cognitive rehabilitation compared to standard of care in treating patients suffering from a stroke in some cognitive domains in a randomized controlled pilot trial. The findings support the further use and study of VR training incorporating activities of daily living in other neurological disorders involving cognitive dysfunction."

INTRODUCTION

2a) In INTRODUCTION: Scientific background and explanation of rationale

2a-i) Problem and the type of system/solution

Describe the problem and the type of system/solution that is object of the study: intended as stand-alone intervention vs. incorporated in broader health care program? Intended for a particular patient population? Goals of the intervention, e.g., being more cost-effective to other interventions, replace or complement other solutions? (Note: Details about the intervention are provided in “Methods” under 5)

subitem not at all important

1 ○

2 ○

3 ○

4 ○

5 ◉

essential

Auswahl löschen

Does your paper address subitem 2a-i? *

Copy and paste relevant sections from the manuscript (include quotes in quotation marks "like this" to indicate direct quotes from your manuscript), or elaborate on this item by providing additional information not in the ms, or briefly explain why the item is not applicable/relevant for your study

"The VR group received treatment with an early version of teora mind, conceptualized and developed by the medical device manufacturer living brain (Heidelberg, Germany), a cognitive training in immersive virtual reality (VRCT). teora mind is a certified medical device of risk class IIa according to the Medical Device Regulation (EU) 2017/745. The certification procedure was executed by the Notified Body TÜV Süd in 2021. The SG integrates ADL and cognitive training methods in a playful way to improve cognition and transfer into daily life and consists of realistic scenarios for training specific abilities. Subjects were trained in a virtual garden and kitchen scenario. In the kitchen, patients were asked to make coffee for multiple people. Therefore, they had to actively implement all necessary steps for serving coffee, including filling the coffee machine with beans and water, changing the coffee filter, choosing a mug, and considering if the coffee has to be served with milk and sugar. Other exercises included sorting groceries according to their categories, e.g., beverages, fruits and vegetables, and durable food for stock, or sorting out the refrigerator according to specifications like spoiled food. A time pressure component was added with increasing difficulty. All activities were implemented in real-time and through patient interaction with the virtual environment."

"The control group received the CCT used in routine care at the rehabilitation center at least three times a week for 30 minutes each session, in this case, Freshminder. Freshminder is a cognitive training program with multiple training paradigms, focusing on attention, planning, and memory. The training is conducted on a PC with a keyboard and a mouse. Users must interact with the application by clicking objects or deciding by pressing a key on the keyboard. In this study, the following tasks were considered: pearlfish (vigilance, working memory), picture series (memory), step sequence (action planning), task switching (flexibility), and double play (divided attention). For example, in the pearlfish task, subjects had to click on fish that carry pearls with them. Subjects had to discriminate which fish were holding pearls and identify the fish at the correct time so that the pearl fell appropriately into the box at the bottom of the sea. This exercise trains selective attention, visual perception, and visuomotor skills. All exercises could be adapted to the patient's performance by changing difficulty and level at the beginning of each task. This was adapted by the therapist in charge. Patients received information about their performance in the training tasks. They could choose from a subset of exercises, with the therapist ensuring that all exercises were performed at a well-balanced frequency."

## 2a-ii) Scientific background, rationale: What is known about the (type of) system

Scientific background, rationale: What is known about the (type of) system that is the object of the study (be sure to discuss the use of similar systems for other conditions/diagnoses, if appropiate), motivation for the study, i.e. what are the reasons for and what is the context for this specific study, from which stakeholder viewpoint is the study performed, potential impact of findings [2]. Briefly justify the choice of the comparator.

subitem not at all important

- 1 ○
- 2 ○
- 3 ○
- 4 ○
- 5 ◉

essential

Auswahl löschen

Does your paper address subitem 2a-ii? *

Copy and paste relevant sections from the manuscript (include quotes in quotation marks "like this" to indicate direct quotes from your manuscript), or elaborate on this item by providing additional information not in the ms, or briefly explain why the item is not applicable/relevant for your study

"Instead, customized virtual reality (VR) training is suitable for multi-domain ADL training [13, 14]. VR is defined as images and sounds created by a computer that seems almost indistinguishable from the real world and can be interacted with by users with sensors [15]. Although it can be experienced nowadays with purpose-made head-mounted displays (HMD) placed on the head in front of the eyes for a fully immersive experience, the interaction with a virtual world is mainly carried out with two hand controllers. VR enables the combination of principles from occupational therapy, neuropsychology, and ADL, resulting in an ecologically valid training setting for many individuals with neurological impairments who want to regain their ability to participate in daily activities. While insights exist concerning the acceptance of immersive HMD-VR in stroke patients [16] or older adults [17], the knowledge about the processes of cognitive rehabilitation facilitated by immersive VR needs to be expanded."

One concept that is becoming more widespread in neurorehabilitation is serious games (SG) [18]. SG are defined as games developed not only for entertainment but also for serious learning purposes and can be considered a non-pharmacological, gamified approach to treat and evaluate [19] a patient's executive dysfunction in an ecologically valid way [20]. SG and VR have already been combined, as Faria et al. demonstrated: They trained EF in immersive VR with a virtual city - Reh@City - including attention, memory, visuospatial abilities, and other EF in performing ADL and found out that this approach boosts cognitive rehabilitation and leads to better results in contrast to conventional cognitive rehabilitation methods [21]. A systematic review of the assessment and treatment of EF with VR in clinical populations and healthy subjects concluded that VR is a promising tool but that more research is necessary, as the authors cite only a few trials on this topic. Training systems focused on ADL hold the potential for better far transfer to the real world after rehabilitation. However, more evidence on the interrelation between SG, ADL, EF training, and immersive VR [22] is necessary.

Investigating VR-based cognitive rehabilitation is where the research of different groups revolves around. The number of publications in scientific journals has increased almost exponentially since 2004 [23]. The three most frequently studied diseases in VR-based cognitive rehabilitation are stroke, dementia, and mild cognitive impairment. Jahn et al. noted in their systematic review of publications about VR and cognitive rehabilitation that there is promising evidence of the functionality of this approach. However, current research findings still struggle with several issues, including group sizes being too small (N=6-34 participants with 89% of reviewed trials having N<20 participants per intervention arm), lack of randomization (e.g., no randomization at all) or suboptimal statistical analyses (no group by time interaction analysis reported) [24].

Maggio et al. found in their review of virtual reality for cognitive rehabilitation that VR training in neurorehabilitation has measurable effects on executive functioning and visuospatial abilities, speech, attention, and memory skills [14]. Particularly executive functioning and depression at discharge from stroke rehabilitation are strong predictors of functioning at the time of discharge and the following 12 months, as Shea-Shumsky et al. found out in their study: Executive functioning was a strong predictor of ADL capacity, to a more significant extent than mental status. It is a strong signal that problems with EF are involved in issues regarding functionality in everyday life and that viable training is helpful and necessary [25]."

2b) In INTRODUCTION: Specific objectives or hypotheses

Does your paper address CONSORT subitem 2b? *

Copy and paste relevant sections from the manuscript (include quotes in quotation marks "like this" to indicate direct quotes from your manuscript), or elaborate on this item by providing additional information not in the ms, or briefly explain why the item is not applicable/relevant for your study

- The primary objective is to assess the impact of an ADL-based VR-SG on patients' cognitive abilities after acute brain affection throughout a 4-week treatment and compared to a conventional CCT treatment.
- The secondary objective is assessing the impact of an ADL-based VR-SG on self-reported outcomes, quality of life, state of health, affect, and compared to a conventional CCT treatment.
- The tertiary objective is to assess the impact of an ADL-based VR-SG on the actual transfer of learned abilities in VR to daily life and reality compared to a conventional CCT treatment.
- The exploratory objective was to investigate the extent to which an ADL-based VR-SG is suitable for cognitive rehabilitation from phase C onwards.

METHODS

3a) Description of trial design (such as parallel, factorial) including allocation ratio

Does your paper address CONSORT subitem 3a? *

Copy and paste relevant sections from the manuscript (include quotes in quotation marks "like this" to indicate direct quotes from your manuscript), or elaborate on this item by providing additional information not in the ms, or briefly explain why the item is not applicable/relevant for your study

"The trial was planned with a parallel design."

3b) Important changes to methods after trial commencement (such as eligibility criteria), with reasons

Does your paper address CONSORT subitem 3b? *

Copy and paste relevant sections from the manuscript (include quotes in quotation marks "like this" to indicate direct quotes from your manuscript), or elaborate on this item by providing additional information not in the ms, or briefly explain why the item is not applicable/relevant for your study

The item is not relevant, as there haven't been changes to methods after trial commencement. If there haven't been changes this has not to be explicitly reported.

3b-i) Bug fixes, Downtimes, Content Changes

Bug fixes, Downtimes, Content Changes: ehealth systems are often dynamic systems. A description of changes to methods therefore also includes important changes made on the intervention or comparator during the trial (e.g., major bug fixes or changes in the functionality or content) (5-iii) and other “unexpected events” that may have influenced study design such as staff changes, system failures/downtimes, etc. [2].

subitem not at all important

- 1 (selected)
- 2
- 3
- 4
- 5

essential

Auswahl löschen

Does your paper address subitem 3b-i?

Copy and paste relevant sections from the manuscript (include quotes in quotation marks "like this" to indicate direct quotes from your manuscript), or elaborate on this item by providing additional information not in the ms, or briefly explain why the item is not applicable/relevant for your study

The item is not relevant, as there haven't been changes to methods after trial commencement. No significant changes to the used software were made while trial conduction. If there haven't been changes this has not to be explicitly reported.

4a) Eligibility criteria for participants

Does your paper address CONSORT subitem 4a? *

Copy and paste relevant sections from the manuscript (include quotes in quotation marks "like this" to indicate direct quotes from your manuscript), or elaborate on this item by providing additional information not in the ms, or briefly explain why the item is not applicable/relevant for your study

"A phase C status qualified patients to participate in the clinical trial, and transition to phase D during study participation was possible. No patient started earlier or later than phase C. All participants were required to be legally competent and of age and had to undergo inpatient neurological rehabilitation treatment at the neurorehabilitation clinic due to acute brain affection (e.g., cerebral infarction, intracerebral hemorrhage, subarachnoid hemorrhage, subdural or epidural intracranial hemorrhage, traumatic brain injury, brain tumor, meningitis or encephalitis, hypoxic encephalopathy), had to have full functionality for at least one arm, known adequate or corrected eyesight in the central field of vision and had to give written consent to participate. Patients were also screened for cognitive performance using the MMST [29]. The obtained score had to be >20 out of 30. The cutoff value was chosen as some cognitive impairment is expected in this sample, and scores of 27-30 (no cognitive impairment) would not properly represent the sample of patients undergoing neurorehabilitation. Patients were excluded if they had present or prior psychiatric comorbidities/disorders and known overreaction to visual stimulation leading to seizures"

4a-i) Computer / Internet literacy

Computer / Internet literacy is often an implicit “de facto” eligibility criterion - this should be explicitly clarified.

subitem not at all important

- ○ 1
- ○ 2
- ● 3
- ○ 4
- ○ 5

essential

Auswahl löschen

Does your paper address subitem 4a-i?

Copy and paste relevant sections from the manuscript (include quotes in quotation marks "like this" to indicate direct quotes from your manuscript), or elaborate on this item by providing additional information not in the ms, or briefly explain why the item is not applicable/relevant for your study

We don't clarify this criterion explicitly, as we introduce a completely new technology to a group of patients that is not in itself familiar with the internet or similar technologies. Therefore we didn't make it a basic requirement.

4a-ii) Open vs. closed, web-based vs. face-to-face assessments:

Open vs. closed, web-based vs. face-to-face assessments: Mention how participants were recruited (online vs. offline), e.g., from an open access website or from a clinic, and clarify if this was a purely web-based trial, or there were face-to-face components (as part of the intervention or for assessment), i.e., to what degree got the study team to know the participant. In online-only trials, clarify if participants were quasi-anonymous and whether having multiple identities was possible or whether technical or logistical measures (e.g., cookies, email confirmation, phone calls) were used to detect/prevent these.

subitem not at all important

- 1 ○
- 2 ○
- 3 ○
- 4 ● (selected)
- 5 ○

essential

Auswahl löschen

Does your paper address subitem 4a-ii? *

Copy and paste relevant sections from the manuscript (include quotes in quotation marks "like this" to indicate direct quotes from your manuscript), or elaborate on this item by providing additional information not in the ms, or briefly explain why the item is not applicable/relevant for your study

"Participants were screened and recruited for enrollment at the Asklepios Neurologische Klinik Falkenstein clinic in Königstein im Taunus, Germany." "The admission interview at the rehabilitation clinic and the patient records of newly admitted rehabilitation patients were used to recruit the sample. A phase C status qualified patients to participate in the clinical trial, and transition to phase D during study participation was possible. No patient started earlier or later than phase C."

4a-iii) Information giving during recruitment

Information given during recruitment. Specify how participants were briefed for recruitment and in the informed consent procedures (e.g., publish the informed consent documentation as appendix, see also item X26), as this information may have an effect on user self-selection, user expectation and may also bias results.

subitem not at all important

1 ○

2 ○

3 ○

4 ○

5 ◉

essential

Auswahl löschen

Does your paper address subitem 4a-iii?

Copy and paste relevant sections from the manuscript (include quotes in quotation marks "like this" to indicate direct quotes from your manuscript), or elaborate on this item by providing additional information not in the ms, or briefly explain why the item is not applicable/relevant for your study

"Once patients eligible for the study had been identified, they were targeted by trained study personnel and informed of the opportunity to participate. In this course, the contents and objectives of the study were explained." - Patients received a form that informed about the whole trial and its targets.

4b) Settings and locations where the data were collected

Does your paper address CONSORT subitem 4b? *

Copy and paste relevant sections from the manuscript (include quotes in quotation marks "like this" to indicate direct quotes from your manuscript), or elaborate on this item by providing additional information not in the ms, or briefly explain why the item is not applicable/relevant for your study

"Participants were screened and recruited for enrollment at the Asklepios Neurologische Klinik Falkenstein clinic in Königstein im Taunus, Germany."

4b-i) Report if outcomes were (self-)assessed through online questionnaires

Clearly report if outcomes were (self-)assessed through online questionnaires (as common in web-based trials) or otherwise.

subitem not at all important

1 ○

2 ○

3 ○

4 ○

5 ○

essential

Does your paper address subitem 4b-i? *

Copy and paste relevant sections from the manuscript (include quotes in quotation marks "like this" to indicate direct quotes from your manuscript), or elaborate on this item by providing additional information not in the ms, or briefly explain why the item is not applicable/relevant for your study

"The testing consisted of psychological tests conducted by a psychologist and self-assessed questionnaires."

4b-ii) Report how institutional affiliations are displayed

Report how institutional affiliations are displayed to potential participants [on ehealth media], as affiliations with prestigious hospitals or universities may affect volunteer rates, use, and reactions with regards to an intervention.(Not a required item – describe only if this may bias results)

subitem not at all important

1 (selected)

2

3

4

5

essential

Auswahl löschen

Does your paper address subitem 4b-ii?

Copy and paste relevant sections from the manuscript (include quotes in quotation marks "like this" to indicate direct quotes from your manuscript), or elaborate on this item by providing additional information not in the ms, or briefly explain why the item is not applicable/relevant for your study

Meine Antwort

5) The interventions for each group with sufficient details to allow replication, including how and when they were actually administered

5-i) Mention names, credential, affiliations of the developers, sponsors, and owners

Mention names, credential, affiliations of the developers, sponsors, and owners [6] (if authors/evaluators are owners or developer of the software, this needs to be declared in a “Conflict of interest” section or mentioned elsewhere in the manuscript).

subitem not at all important

1 ○

2 ○

3 ◉

4 ○

5 ○

essential

Auswahl löschen

Does your paper address subitem 5-i?

Copy and paste relevant sections from the manuscript (include quotes in quotation marks "like this" to indicate direct quotes from your manuscript), or elaborate on this item by providing additional information not in the ms, or briefly explain why the item is not applicable/relevant for your study

"This work was partially funded by the medical device manufacturer living brain GmbH, Heidelberg, DE, who developed the used VR software. The co-authors of this publication, Barbara Stegmann and Julian Specht are the co-founders of living brain."

### 5-ii) Describe the history/development process

Describe the history/development process of the application and previous formative evaluations (e.g., focus groups, usability testing), as these will have an impact on adoption/use rates and help with interpreting results.

subitem not at all important

- 1 ○
- 2 ○
- 3 ◉
- 4 ○
- 5 ○

essential

Auswahl löschen

### Does your paper address subitem 5-ii?

Copy and paste relevant sections from the manuscript (include quotes in quotation marks "like this" to indicate direct quotes from your manuscript), or elaborate on this item by providing additional information not in the ms, or briefly explain why the item is not applicable/relevant for your study

"Overall, acceptance and usability of both technologies were reportedly observable high, which is concerning the VR experience congruent to the results of our feasibility study with an earlier version of the software used in this trial [16]."

### 5-iii) Revisions and updating

Revisions and updating. Clearly mention the date and/or version number of the application/intervention (and comparator, if applicable) evaluated, or describe whether the intervention underwent major changes during the evaluation process, or whether the development and/or content was “frozen” during the trial. Describe dynamic components such as news feeds or changing content which may have an impact on the replicability of the intervention (for unexpected events see item 3b).

subitem not at all important

- 1 ○
- 2 ○
- 3 ◉
- 4 ○
- 5 ○

essential

Auswahl löschen

### Does your paper address subitem 5-iii?

Copy and paste relevant sections from the manuscript (include quotes in quotation marks "like this" to indicate direct quotes from your manuscript), or elaborate on this item by providing additional information not in the ms, or briefly explain why the item is not applicable/relevant for your study

In the trial, an early, specifically for this clinical trial, prepared version has been used. At that time, the medical device had not been introduced to the market. It was updated two times in the course of the trial for minor bugs.

5-iv) Quality assurance methods

Provide information on quality assurance methods to ensure accuracy and quality of information provided [1], if applicable.

subitem not at all important

1 ○

2 ○

3 ○

4 ○

5 ○

essential

Does your paper address subitem 5-iv?

Copy and paste relevant sections from the manuscript (include quotes in quotation marks "like this" to indicate direct quotes from your manuscript), or elaborate on this item by providing additional information not in the ms, or briefly explain why the item is not applicable/relevant for your study

Meine Antwort

5-v) Ensure replicability by publishing the source code, and/or providing screenshots/screen-capture video, and/or providing flowcharts of the algorithms used

Ensure replicability by publishing the source code, and/or providing screenshots/screen-capture video, and/or providing flowcharts of the algorithms used. Replicability (i.e., other researchers should in principle be able to replicate the study) is a hallmark of scientific reporting.

subitem not at all important

1 ○

2 ○

3 ◉

4 ○

5 ○

essential

Auswahl löschen

Does your paper address subitem 5-v?

Copy and paste relevant sections from the manuscript (include quotes in quotation marks "like this" to indicate direct quotes from your manuscript), or elaborate on this item by providing additional information not in the ms, or briefly explain why the item is not applicable/relevant for your study

Pictures of the applications used in this trial are available in the appendix and the methods section.

### 5-vi) Digital preservation

Digital preservation: Provide the URL of the application, but as the intervention is likely to change or disappear over the course of the years; also make sure the intervention is archived (Internet Archive, webcitation.org, and/or publishing the source code or screenshots/videos alongside the article). As pages behind login screens cannot be archived, consider creating demo pages which are accessible without login.

subitem not at all important

- 1 (selected)
- 2
- 3
- 4
- 5

essential

Auswahl löschen

### Does your paper address subitem 5-vi?

Copy and paste relevant sections from the manuscript (include quotes in quotation marks "like this" to indicate direct quotes from your manuscript), or elaborate on this item by providing additional information not in the ms, or briefly explain why the item is not applicable/relevant for your study

As the applications used in this trial are commercialized, it's impossible to archive the source code so that other researchers can use it.

## 5-vii) Access

Access: Describe how participants accessed the application, in what setting/context, if they had to pay (or were paid) or not, whether they had to be a member of specific group. If known, describe how participants obtained “access to the platform and Internet” [1]. To ensure access for editors/reviewers/readers, consider to provide a “backdoor” login account or demo mode for reviewers/readers to explore the application (also important for archiving purposes, see vi).

subitem not at all important

1 ○

2 ○

3 ○

4 ○

5 ○

essential

## Does your paper address subitem 5-vii? *

Copy and paste relevant sections from the manuscript (include quotes in quotation marks "like this" to indicate direct quotes from your manuscript), or elaborate on this item by providing additional information not in the ms, or briefly explain why the item is not applicable/relevant for your study

"The trial compares two treatments: a novel immersive VR intervention to treat cognitive dysfunction, specifically EF, presenting ADL in the form of an SG, and conventional computerized cognitive rehabilitation training in inpatient care at a German rehabilitation hospital."

"All participants were required to be legally competent and of age and had to undergo inpatient neurological rehabilitation treatment at the neurorehabilitation clinic due to acute brain affection (e.g., cerebral infarction,...)"

By the nature of the patient group (patients in inpatient neurological rehabilitation), their only access to the solutions was by staying at the hospital.

### 5-viii) Mode of delivery, features/functionalities/components of the intervention and comparator, and the theoretical framework

Describe mode of delivery, features/functionalities/components of the intervention and comparator, and the theoretical framework [6] used to design them (instructional strategy [1], behaviour change techniques, persuasive features, etc., see e.g., [7, 8] for terminology). This includes an in-depth description of the content (including where it is coming from and who developed it) [1],” whether [and how] it is tailored to individual circumstances and allows users to track their progress and receive feedback” [6]. This also includes a description of communication delivery channels and – if computer-mediated communication is a component – whether communication was synchronous or asynchronous [6]. It also includes information on presentation strategies [1], including page design principles, average amount of text on pages, presence of hyperlinks to other resources, etc. [1].

subitem not at all important

1 ○

2 ○

3 ○

4 ○

5 ○

essential

Does your paper address subitem 5-viii? *

Copy and paste relevant sections from the manuscript (include quotes in quotation marks "like this" to indicate direct quotes from your manuscript), or elaborate on this item by providing additional information not in the ms, or briefly explain why the item is not applicable/relevant for your study

"The VR group received treatment with an early version of teora mind, conceptualized and developed by the medical device manufacturer living brain (Heidelberg, Germany), a cognitive training in immersive virtual reality (VRCT). teora mind is a certified medical device of risk class IIa according to the Medical Device Regulation (EU) 2017/745. The certification procedure was executed by the Notified Body TÜV Süd in 2021. The SG integrates ADL and cognitive training methods in a playful way to improve cognition and transfer into daily life and consists of realistic scenarios for training specific abilities. Subjects were trained in a virtual garden and kitchen scenario. In the kitchen, patients were asked to make coffee for multiple people. Therefore, they had to actively implement all necessary steps for serving coffee, including filling the coffee machine with beans and water, changing the coffee filter, choosing a mug, and considering if the coffee has to be served with milk and sugar. Other exercises included sorting groceries according to their categories, e.g., beverages, fruits and vegetables, and durable food for stock, or sorting out the refrigerator according to specifications like spoiled food. A time pressure component was added with increasing difficulty. All activities were implemented in real-time and through patient interaction with the virtual environment.
In the garden scenario, patients were asked to plant seeds for growing strawberries, tomatoes, and cucumbers. In the growth phase, users had to ensure that there was always enough water in the bed and that popping weeds did not destroy the seedlings, which required watering the bed with a watering can. After successfully growing fruits and vegetables, users could produce juices from the harvested food. Subjects had to plan their activity by first sorting picture cards describing each action step on a board in what they thought was the correct order. Next, they had to confirm the order. Finally, the system reported whether the order was correct or incorrect and indicated which steps were arranged incorrectly. Only after the right order was achieved, the exercise could begin. The complexity varied depending on the difficulty level; at the highest level, planning was omitted, and the activity had to be started immediately.

The main cognitive domains targeted by the VR training are EFs, thereof action planning, problem-solving, selective attention, and working memory. The specific pieces of training themselves do not primarily focus on a single cognitive domain but rather on training the ADL, which typically comprises multiple cognitive domains simultaneously. Like the CCT intervention, the difficulty could be adapted to the individual performance by changing to 20 different degrees of difficulty. The therapist in charge adapted this. The patients received information in the VR application about how they performed their training tasks. In the trial, an early, specifically for this clinical trial, prepared version has been used. At that time, the medical device had not been introduced to the market. It was updated two times in the course of the trial for minor bugs.

VR devices of the brand Oculus® Quest (Meta) were used for trial conduction. The device is a standalone HMD, further technical devices for experiencing immersive VR are not required. In addition to the VR device, the study nurses and therapists in charge received a tablet (Samsung® Galaxy Tab A) for monitoring ongoing VR activities; all VR procedures were mirrored on the tablet in real time. This allowed patient-therapist interaction despite the high grade of immersion and was particularly in the first training sessions of benefit. Interaction in immersive VR was enabled by hand controllers patients used. Blue hands visually represented them and were congruent with the actual movement of the upper extremities. The controllers included multiple buttons, of which only two were necessary for using this VR software. Moving the head made it possible to gain a complete overview of the virtual world. Through a visible white beam, users could assess what they were currently pointing at in the virtual environment. This allowed the selection of objects. To introduce patients to the technology and the virtual environment, they went through a dedicated tutorial at the beginning of each treatment."

[illegible]

### 5-ix) Describe use parameters

Describe use parameters (e.g., intended “doses” and optimal timing for use). Clarify what instructions or recommendations were given to the user, e.g., regarding timing, frequency, heaviness of use, if any, or was the intervention used ad libitum.

subitem not at all important

- 1 ( )
- 2 ( )
- 3 ( )
- 4 ( )
- 5 (x)

essential

Auswahl löschen

Does your paper address subitem 5-ix?

Copy and paste relevant sections from the manuscript (include quotes in quotation marks "like this" to indicate direct quotes from your manuscript), or elaborate on this item by providing additional information not in the ms, or briefly explain why the item is not applicable/relevant for your study

"The treatment was scheduled for 18-25 sessions with a duration of 30 minutes for the individual, based on the date of admission and the participant's immediate health and neurological condition. The sessions were conducted at least three to five days a week. Both groups received their allocated treatment from day 3 to at least day 20. A maximum of 25 treatment sessions was allowed."

"Participants in this trial received a treatment dosage of 18.7 sessions over 4-6 weeks, each lasting 30 minutes (9.35h per participant). Other trials reported an average intervention duration of 13.3 hours [40, 48, 49, 50, 51, 52], ranging from 4 to 24 hours, with a session duration of 30 to 60 minutes, 3 to 5 times a week. Our dosing was sufficient to achieve a measurable effect in some domains and was also oriented on the care reality in German rehabilitation hospitals. For attaining more significant outcomes in multiple domains, it would be interesting to see if other training tasks in immersive VR create an effect on cognition and if a varying number of treatment sessions beyond inpatient care leads to different results than those reported here. Based on the results of this trial, we conclude that 18-19 sessions à 30 minutes are sufficient to achieve a measurable treatment effect. Still, we cannot answer whether fewer sessions would create similar effects, if more treatment automatically leads to more significant outcomes, and if those effects vary between patient groups. More extensive trials with blinding, subgroups, and longer treatment duration are necessary to answer this question."

### 5-x) Clarify the level of human involvement

Clarify the level of human involvement (care providers or health professionals, also technical assistance) in the e-intervention or as co-intervention (detail number and expertise of professionals involved, if any, as well as “type of assistance offered, the timing and frequency of the support, how it is initiated, and the medium by which the assistance is delivered”. It may be necessary to distinguish between the level of human involvement required for the trial, and the level of human involvement required for a routine application outside of a RCT setting (discuss under item 21 – generalizability).

subitem not at all important

- 1 ○
- 2 ○
- 3 ◉
- 4 ○
- 5 ○

essential

Auswahl löschen

### Does your paper address subitem 5-x?

Copy and paste relevant sections from the manuscript (include quotes in quotation marks "like this" to indicate direct quotes from your manuscript), or elaborate on this item by providing additional information not in the ms, or briefly explain why the item is not applicable/relevant for your study

"The study nurse instructed the intervention group on the device’s functions and use before the first VR training session." "During all training sessions, the study nurse was able to track the process on a tablet and was thus able to provide accurate, customized assistance if needed."

5-xi) Report any prompts/reminders used

Report any prompts/reminders used: Clarify if there were prompts (letters, emails, phone calls, SMS) to use the application, what triggered them, frequency etc. It may be necessary to distinguish between the level of prompts/reminders required for the trial, and the level of prompts/reminders for a routine application outside of a RCT setting (discuss under item 21 – generalizability).

subitem not at all important

- 1 (selected)
- 2
- 3
- 4
- 5

essential

Auswahl löschen

Does your paper address subitem 5-xi? *

Copy and paste relevant sections from the manuscript (include quotes in quotation marks "like this" to indicate direct quotes from your manuscript), or elaborate on this item by providing additional information not in the ms, or briefly explain why the item is not applicable/relevant for your study

No prompts or reminders were used. It was not relevant as the software was used in attendance of a study nurse.

### 5-xii) Describe any co-interventions (incl. training/support)

Describe any co-interventions (incl. training/support): Clearly state any interventions that are provided in addition to the targeted eHealth intervention, as ehealth intervention may not be designed as stand-alone intervention. This includes training sessions and support [1]. It may be necessary to distinguish between the level of training required for the trial, and the level of training for a routine application outside of a RCT setting (discuss under item 21 – generalizability.

subitem not at all important

1 ○

2 ○

3 ●

4 ○

5 ○

essential

Auswahl löschen

### Does your paper address subitem 5-xii? *

Copy and paste relevant sections from the manuscript (include quotes in quotation marks "like this" to indicate direct quotes from your manuscript), or elaborate on this item by providing additional information not in the ms, or briefly explain why the item is not applicable/relevant for your study

The study nurse instructed the intervention group on the device's functions and use before the first VR training session. This included the general operation of the device from the outside with the attached buttons (on-off button, volume control), but especially the handling of the hand controllers. After receiving the instructions, participants went through a VR tutorial, consisting of training in basic functionality, the memory game, and the recycling game, in which they were familiarized with general handling in the first training session. This tutorial demonstrated the interaction with the VR environment, virtual objects, and handling the whole experience. The tutorial was developed and tested with stroke patients, leading to high acceptance in a group of stroke patients and healthy older adults, indicating that it's suited for introducing this critical user group to immersive VR [16]. In addition, the familiarization phase helps to reduce possible effects due to unawareness about the technology and negative attitudes towards it [30]. During all training sessions, the study nurse was able to track the process on a tablet and was thus able to provide accurate, customized assistance if needed.

6a) Completely defined pre-specified primary and secondary outcome measures, including how and when they were assessed

Does your paper address CONSORT subitem 6a? *

Copy and paste relevant sections from the manuscript (include quotes in quotation marks "like this" to indicate direct quotes from your manuscript), or elaborate on this item by providing additional information not in the ms, or briefly explain why the item is not applicable/relevant for your study

- The primary objective is to assess the impact of an ADL-based VR-SG on patients' cognitive abilities after acute brain affection throughout a 4-week treatment and compared to a conventional CCT treatment.
- The secondary objective is assessing the impact of an ADL-based VR-SG on self-reported outcomes, quality of life, state of health, affect, and compared to a conventional CCT treatment.
- The tertiary objective is to assess the impact of an ADL-based VR-SG on the actual transfer of learned abilities in VR to daily life and reality compared to a conventional CCT treatment.
- The exploratory objective was to investigate the extent to which an ADL-based VR-SG is suitable for cognitive rehabilitation from phase C onwards.

6a-i) Online questionnaires: describe if they were validated for online use and apply CHERRIES items to describe how the questionnaires were designed/deployed

If outcomes were obtained through online questionnaires, describe if they were validated for online use and apply CHERRIES items to describe how the questionnaires were designed/deployed [9].

subitem not at all important

- 1 (selected)
- 2
- 3
- 4
- 5

essential

Auswahl löschen

Does your paper address subitem 6a-i?

Copy and paste relevant sections from manuscript text

Meine Antwort

6a-ii) Describe whether and how “use” (including intensity of use/dosage) was defined/measured/monitored

Describe whether and how “use” (including intensity of use/dosage) was defined/measured/monitored (logins, logfile analysis, etc.). Use/adoption metrics are important process outcomes that should be reported in any ehealth trial.

subitem not at all important

1 ○

2 ○

3 ○

4 ◉

5 ○

essential

Auswahl löschen

Does your paper address subitem 6a-ii?

Copy and paste relevant sections from manuscript text

Participants in this trial received a treatment dosage of 18.7 sessions over 4-6 weeks, each lasting 30 minutes (9.35h per participant). Other trials reported an average intervention duration of 13.3 hours [40, 48, 49, 50, 51, 52], ranging from 4 to 24 hours, with a session duration of 30 to 60 minutes, 3 to 5 times a week. Our dosing was sufficient to achieve a measurable effect in some domains and was also oriented on the care reality in German rehabilitation hospitals. For attaining more significant outcomes in multiple domains, it would be interesting to see if other training tasks in immersive VR create an effect on cognition and if a varying number of treatment sessions beyond inpatient care leads to different results than those reported here. Based on the results of this trial, we conclude that 18-19 sessions à 30 minutes are sufficient to achieve a measurable treatment effect. Still, we cannot answer whether fewer sessions would create similar effects, if more treatment automatically leads to more significant outcomes, and if those effects vary between patient groups. More extensive trials with blinding, subgroups, and longer treatment duration are necessary to answer this question.

6a-iii) Describe whether, how, and when qualitative feedback from participants was obtained

Describe whether, how, and when qualitative feedback from participants was obtained (e.g., through emails, feedback forms, interviews, focus groups).

subitem not at all important

1 ○

2 ○

3 ○

4 ○

5 ○

essential

Does your paper address subitem 6a-iii?

Copy and paste relevant sections from manuscript text

Meine Antwort

6b) Any changes to trial outcomes after the trial commenced, with reasons

Does your paper address CONSORT subitem 6b? *

Copy and paste relevant sections from the manuscript (include quotes in quotation marks "like this" to indicate direct quotes from your manuscript), or elaborate on this item by providing additional information not in the ms, or briefly explain why the item is not applicable/relevant for your study

No changes to these objectives were made after trial commencement.

7a) How sample size was determined

NPT: When applicable, details of whether and how the clustering by care provides or centers was addressed

7a-i) Describe whether and how expected attrition was taken into account when calculating the sample size

Describe whether and how expected attrition was taken into account when calculating the sample size.

subitem not at all important

1 ○

2 ○

3 ○

4 ○

5 ○

essential

Does your paper address subitem 7a-i?

Copy and paste relevant sections from manuscript title (include quotes in quotation marks "like this" to indicate direct quotes from your manuscript), or elaborate on this item by providing additional information not in the ms, or briefly explain why the item is not applicable/relevant for your study

Meine Antwort

7b) When applicable, explanation of any interim analyses and stopping guidelines

Does your paper address CONSORT subitem 7b? *

Copy and paste relevant sections from the manuscript (include quotes in quotation marks "like this" to indicate direct quotes from your manuscript), or elaborate on this item by providing additional information not in the ms, or briefly explain why the item is not applicable/relevant for your study

No interim analyses have been performed, and no interim analyses have been planned before the trial start.

8a) Method used to generate the random allocation sequence

NPT: When applicable, how care providers were allocated to each trial group

Does your paper address CONSORT subitem 8a? *

Copy and paste relevant sections from the manuscript (include quotes in quotation marks "like this" to indicate direct quotes from your manuscript), or elaborate on this item by providing additional information not in the ms, or briefly explain why the item is not applicable/relevant for your study

The process of participant randomization is described in the flowchart prepared according to the CONSORT guidelines, we used an analogue randomization process. Simple randomization was done by the study nurse.

8b) Type of randomisation; details of any restriction (such as blocking and block size)

Does your paper address CONSORT subitem 8b? *

Copy and paste relevant sections from the manuscript (include quotes in quotation marks "like this" to indicate direct quotes from your manuscript), or elaborate on this item by providing additional information not in the ms, or briefly explain why the item is not applicable/relevant for your study

The process of participant randomization is described in the flowchart prepared according to the CONSORT guidelines, we used an analog randomization process by drawing notes from an urn. Simple randomization was done by the study nurse.

9) Mechanism used to implement the random allocation sequence (such as sequentially numbered containers), describing any steps taken to conceal the sequence until interventions were assigned

Does your paper address CONSORT subitem 9? *

Copy and paste relevant sections from the manuscript (include quotes in quotation marks "like this" to indicate direct quotes from your manuscript), or elaborate on this item by providing additional information not in the ms, or briefly explain why the item is not applicable/relevant for your study

The process of participant randomization is described in the flowchart prepared according to the CONSORT guidelines, we used an analog randomization process by drawing notes from an urn

10) Who generated the random allocation sequence, who enrolled participants, and who assigned participants to interventions

Does your paper address CONSORT subitem 10? *

Copy and paste relevant sections from the manuscript (include quotes in quotation marks "like this" to indicate direct quotes from your manuscript), or elaborate on this item by providing additional information not in the ms, or briefly explain why the item is not applicable/relevant for your study

Simple randomization was done by the study nurse.

11a) If done, who was blinded after assignment to interventions (for example, participants, care providers, those assessing outcomes) and how

NPT: Whether or not administering co-interventions were blinded to group assignment

11a-i) Specify who was blinded, and who wasn't

Specify who was blinded, and who wasn't. Usually, in web-based trials it is not possible to blind the participants [1, 3] (this should be clearly acknowledged), but it may be possible to blind outcome assessors, those doing data analysis or those administering co-interventions (if any).

subitem not at all important

1 ○

2 ○

3 ○

4 ○

5 ○

essential

Does your paper address subitem 11a-i? *

Copy and paste relevant sections from the manuscript (include quotes in quotation marks "like this" to indicate direct quotes from your manuscript), or elaborate on this item by providing additional information not in the ms, or briefly explain why the item is not applicable/relevant for your study

The trial was not blinded.

11a-ii) Discuss e.g., whether participants knew which intervention was the "intervention of interest" and which one was the "comparator"

Informed consent procedures (4a-ii) can create biases and certain expectations - discuss e.g., whether participants knew which intervention was the "intervention of interest" and which one was the "comparator".

subitem not at all important

1 ○

2 ○

3 ○

4 ○

5 ○

essential

Does your paper address subitem 11a-ii?

Copy and paste relevant sections from the manuscript (include quotes in quotation marks "like this" to indicate direct quotes from your manuscript), or elaborate on this item by providing additional information not in the ms, or briefly explain why the item is not applicable/relevant for your study

Meine Antwort

11b) If relevant, description of the similarity of interventions

(this item is usually not relevant for ehealth trials as it refers to similarity of a placebo or sham intervention to a active medication/intervention)

Does your paper address CONSORT subitem 11b? *

Copy and paste relevant sections from the manuscript (include quotes in quotation marks "like this" to indicate direct quotes from your manuscript), or elaborate on this item by providing additional information not in the ms, or briefly explain why the item is not applicable/relevant for your study

The differences on the interventions became clear when the study nurse introduced participants to the allocated interventions. Also were patients often on the same stations and had the chance to discuss the treatment they currently experience. The treatments are described in the methods section.

12a) Statistical methods used to compare groups for primary and secondary outcomes

NPT: When applicable, details of whether and how the clustering by care providers or centers was addressed

Does your paper address CONSORT subitem 12a? *

Copy and paste relevant sections from the manuscript (include quotes in quotation marks "like this" to indicate direct quotes from your manuscript), or elaborate on this item by providing additional information not in the ms, or briefly explain why the item is not applicable/relevant for your study

Statistical analyses were run on IBM SPSS 29, considering P<.05 as significant. We calculated repeated measures ANOVA and posthoc tests to detect inner-subject and between-group changes between t0 and t1.
Comparing the VR group and control group in cognitive performance and health-related measures involves group mean comparisons from two independent samples. Power calculations (power analysis tool GPower, release 3.1.9.6 for Mac OS X) revealed that our sample size of N=21 per group was sufficient to detect a standardized estimated population mean difference of f=.25, which is considered a medium effect size, with a power of 1-β=.88 for α=.05. This shows that our small sample size does not constrain detection of between-group effects at a conventional power goal of .8 with a medium effect size of f=.25.

### 12a-i) Imputation techniques to deal with attrition / missing values

Imputation techniques to deal with attrition / missing values: Not all participants will use the intervention/comparator as intended and attrition is typically high in ehealth trials. Specify how participants who did not use the application or dropped out from the trial were treated in the statistical analysis (a complete case analysis is strongly discouraged, and simple imputation techniques such as LOCF may also be problematic [4]).

subitem not at all important

1 ○

2 ○

3 ○

4 ○

5 ○

essential

### Does your paper address subitem 12a-i? *

Copy and paste relevant sections from the manuscript (include quotes in quotation marks "like this" to indicate direct quotes from your manuscript), or elaborate on this item by providing additional information not in the ms, or briefly explain why the item is not applicable/relevant for your study

Only a very few items were missing, so no imputation techniques were needed to apply.

"Of the patients who completed the study, 41 out of 42 participants (97%) completed all questionnaires and tests, with only occasional missing items or tests. This might be explained by the fact that all surveys were conducted in the attendance of a therapist or neuropsychologist working and trained in the clinic."

### 12b) Methods for additional analyses, such as subgroup analyses and adjusted analyses

Does your paper address CONSORT subitem 12b? *

Copy and paste relevant sections from the manuscript (include quotes in quotation marks "like this" to indicate direct quotes from your manuscript), or elaborate on this item by providing additional information not in the ms, or briefly explain why the item is not applicable/relevant for your study

No subgroup or adjusted analyses were conducted, so there is no section about these topics.

X26) REB/IRB Approval and Ethical Considerations [recommended as subheading under "Methods"] (not a CONSORT item)

X26-i) Comment on ethics committee approval

subitem not at all important

1 ○

2 ○

3 ○

4 ○

5 ○

essential

Does your paper address subitem X26-i?

Copy and paste relevant sections from the manuscript (include quotes in quotation marks "like this" to indicate direct quotes from your manuscript), or elaborate on this item by providing additional information not in the ms, or briefly explain why the item is not applicable/relevant for your study

"Approval for this pilot trial was obtained through the local Ethics Committee of the Regional Medical Association Hessen, Germany, under the approval number 2020-1768-fMP. The VR-training teora mind is a certified medical device (class IIa) in line with the European medical device regulation MDR and therefore obtained approval to conduct this trial from the state authority Bundesinstitut für Arzneimittel und Medizinprodukte under the file number 94208-5660-12543. All regulatory approvals were obtained before the recruitment of the first patient."

x26-ii) Outline informed consent procedures

Outline informed consent procedures e.g., if consent was obtained offline or online (how? Checkbox, etc.?), and what information was provided (see 4a-ii). See [6] for some items to be included in informed consent documents.

subitem not at all important

1 ○

2 ○

3 ○

4 ○

5 ○

essential

Does your paper address subitem X26-ii?

Copy and paste relevant sections from the manuscript (include quotes in quotation marks "like this" to indicate direct quotes from your manuscript), or elaborate on this item by providing additional information not in the ms, or briefly explain why the item is not applicable/relevant for your study

Meine Antwort

### X26-iii) Safety and security procedures

Safety and security procedures, incl. privacy considerations, and any steps taken to reduce the likelihood or detection of harm (e.g., education and training, availability of a hotline)

subitem not at all important

1 ○

2 ○

3 ○

4 ○

5 ○

essential

### Does your paper address subitem X26-iii?

Copy and paste relevant sections from the manuscript (include quotes in quotation marks "like this" to indicate direct quotes from your manuscript), or elaborate on this item by providing additional information not in the ms, or briefly explain why the item is not applicable/relevant for your study

"To introduce patients to the technology and the virtual environment, they went through a dedicated tutorial at the beginning of each treatment. "

## RESULTS

### 13a) For each group, the numbers of participants who were randomly assigned, received intended treatment, and were analysed for the primary outcome

NPT: The number of care providers or centers performing the intervention in each group and the number of patients treated by each care provider in each center

Does your paper address CONSORT subitem 13a? *

Copy and paste relevant sections from the manuscript (include quotes in quotation marks "like this" to indicate direct quotes from your manuscript), or elaborate on this item by providing additional information not in the ms, or briefly explain why the item is not applicable/relevant for your study

"Twenty-one patients received the novel VR treatment, the other 21 received the control CCT."

13b) For each group, losses and exclusions after randomisation, together with reasons

Does your paper address CONSORT subitem 13b? (NOTE: Preferably, this is shown in a CONSORT flow diagram) *

Copy and paste relevant sections from the manuscript (include quotes in quotation marks "like this" to indicate direct quotes from your manuscript), or elaborate on this item by providing additional information not in the ms, or briefly explain why the item is not applicable/relevant for your study

"However, 31 were not able to complete the minimum treatment number due to the following reasons: COVID (4), early dismissal (12), psychiatric comorbidities not detected earlier (3), treatment discontinuation due to a high incidence of COVID cases at the trial center (12)."

13b-i) Attrition diagram

Strongly recommended: An attrition diagram (e.g., proportion of participants still logging in or using the intervention/comparator in each group plotted over time, similar to a survival curve) or other figures or tables demonstrating usage/dose/engagement.

subitem not at all important

1 ○

2 ○

3 ○

4 ○

5 ○

essential

Does your paper address subitem 13b-i?

Copy and paste relevant sections from the manuscript or cite the figure number if applicable (include quotes in quotation marks "like this" to indicate direct quotes from your manuscript), or elaborate on this item by providing additional information not in the ms, or briefly explain why the item is not applicable/relevant for your study

Meine Antwort

14a) Dates defining the periods of recruitment and follow-up

Does your paper address CONSORT subitem 14a? *

Copy and paste relevant sections from the manuscript (include quotes in quotation marks "like this" to indicate direct quotes from your manuscript), or elaborate on this item by providing additional information not in the ms, or briefly explain why the item is not applicable/relevant for your study

"The recruitment process started on November 1st 2020, the last patient finished treatment on March 31st 2022."

14a-i) Indicate if critical “secular events” fell into the study period

Indicate if critical “secular events” fell into the study period, e.g., significant changes in Internet resources available or “changes in computer hardware or Internet delivery resources”

subitem not at all important

1 ○

2 ○

3 ○

4 ○

5 ○

essential

Does your paper address subitem 14a-i?

Copy and paste relevant sections from the manuscript (include quotes in quotation marks "like this" to indicate direct quotes from your manuscript), or elaborate on this item by providing additional information not in the ms, or briefly explain why the item is not applicable/relevant for your study

Meine Antwort

14b) Why the trial ended or was stopped (early)

Does your paper address CONSORT subitem 14b? *

Copy and paste relevant sections from the manuscript (include quotes in quotation marks "like this" to indicate direct quotes from your manuscript), or elaborate on this item by providing additional information not in the ms, or briefly explain why the item is not applicable/relevant for your study

"A further extension of the trial was not possible for administrative reasons."

15) A table showing baseline demographic and clinical characteristics for each group

NPT: When applicable, a description of care providers (case volume, qualification, expertise, etc.) and centers (volume) in each group

Does your paper address CONSORT subitem 15? *

Copy and paste relevant sections from the manuscript (include quotes in quotation marks "like this" to indicate direct quotes from your manuscript), or elaborate on this item by providing additional information not in the ms, or briefly explain why the item is not applicable/relevant for your study

The table concerning this topic are Tables 1-3.

15-i) Report demographics associated with digital divide issues

In ehealth trials it is particularly important to report demographics associated with digital divide issues, such as age, education, gender, social-economic status, computer/Internet/ehealth literacy of the participants, if known.

subitem not at all important

- 1 (selected)
- 2
- 3
- 4
- 5

essential

Auswahl löschen

Does your paper address subitem 15-i? *

Copy and paste relevant sections from the manuscript (include quotes in quotation marks "like this" to indicate direct quotes from your manuscript), or elaborate on this item by providing additional information not in the ms, or briefly explain why the item is not applicable/relevant for your study

Since this study refers to a defined target group and participation is not from the total population and additionally takes place in a closed setting (hospital), this item is not applicable.

16) For each group, number of participants (denominator) included in each analysis and whether the analysis was by original assigned groups

16-i) Report multiple “denominators” and provide definitions

Report multiple “denominators” and provide definitions: Report N’s (and effect sizes) “across a range of study participation [and use] thresholds” [1], e.g., N exposed, N consented, N used more than x times, N used more than y weeks, N participants “used” the intervention/comparator at specific pre-defined time points of interest (in absolute and relative numbers per group). Always clearly define “use” of the intervention.

subitem not at all important

1 ○

2 ○

3 ○

4 ○

5 ○

essential

Does your paper address subitem 16-i? *

Copy and paste relevant sections from the manuscript (include quotes in quotation marks "like this" to indicate direct quotes from your manuscript), or elaborate on this item by providing additional information not in the ms, or briefly explain why the item is not applicable/relevant for your study

"...revealed that our sample size of N=21 per group was sufficient to detect a standardized estimated population mean difference of f=.25, which is considered a medium effect size, with a power of 1-β=.88 for α=.05. This shows that our small sample size does not constrain detection of between-group effects at a conventional power goal of .8 with a medium effect size of f=.25."

16-ii) Primary analysis should be intent-to-treat

Primary analysis should be intent-to-treat, secondary analyses could include comparing only “users”, with the appropriate caveats that this is no longer a randomized sample (see 18-i).

subitem not at all important

1 ○

2 ○

3 ○

4 ○

5 ○

essential

Does your paper address subitem 16-ii?

Copy and paste relevant sections from the manuscript (include quotes in quotation marks "like this" to indicate direct quotes from your manuscript), or elaborate on this item by providing additional information not in the ms, or briefly explain why the item is not applicable/relevant for your study

Meine Antwort

17a) For each primary and secondary outcome, results for each group, and the estimated effect size and its precision (such as 95% confidence interval)

Does your paper address CONSORT subitem 17a? *

Copy and paste relevant sections from the manuscript (include quotes in quotation marks "like this" to indicate direct quotes from your manuscript), or elaborate on this item by providing additional information not in the ms, or briefly explain why the item is not applicable/relevant for your study

All results are demonstrated in Table 4.

17a-i) Presentation of process outcomes such as metrics of use and intensity of use

In addition to primary/secondary (clinical) outcomes, the presentation of process outcomes such as metrics of use and intensity of use (dose, exposure) and their operational definitions is critical. This does not only refer to metrics of attrition (13-b) (often a binary variable), but also to more continuous exposure metrics such as “average session length”. These must be accompanied by a technical description how a metric like a “session” is defined (e.g., timeout after idle time) [1] (report under item 6a).

subitem not at all important

1 ○

2 ○

3 ○

4 ○

5 ○

essential

Does your paper address subitem 17a-i?

Copy and paste relevant sections from the manuscript (include quotes in quotation marks "like this" to indicate direct quotes from your manuscript), or elaborate on this item by providing additional information not in the ms, or briefly explain why the item is not applicable/relevant for your study

Meine Antwort

17b) For binary outcomes, presentation of both absolute and relative effect sizes is recommended

Does your paper address CONSORT subitem 17b? *

Copy and paste relevant sections from the manuscript (include quotes in quotation marks "like this" to indicate direct quotes from your manuscript), or elaborate on this item by providing additional information not in the ms, or briefly explain why the item is not applicable/relevant for your study

This section is not applicable, as there are no binary outcomes, the outcomes of this trial are multi-faceted, and therefore a relative effect size is not described.

18) Results of any other analyses performed, including subgroup analyses and adjusted analyses, distinguishing pre-specified from exploratory

Does your paper address CONSORT subitem 18? *

Copy and paste relevant sections from the manuscript (include quotes in quotation marks "like this" to indicate direct quotes from your manuscript), or elaborate on this item by providing additional information not in the ms, or briefly explain why the item is not applicable/relevant for your study

There are no other analyses besides those described in the study rationale.

### 18-i) Subgroup analysis of comparing only users

A subgroup analysis of comparing only users is not uncommon in ehealth trials, but if done, it must be stressed that this is a self-selected sample and no longer an unbiased sample from a randomized trial (see 16-iii).

subitem not at all important

1 ○

2 ○

3 ○

4 ○

5 ○

essential

### Does your paper address subitem 18-i?

Copy and paste relevant sections from the manuscript (include quotes in quotation marks "like this" to indicate direct quotes from your manuscript), or elaborate on this item by providing additional information not in the ms, or briefly explain why the item is not applicable/relevant for your study

Meine Antwort

### 19) All important harms or unintended effects in each group

(for specific guidance see CONSORT for harms)

Does your paper address CONSORT subitem 19? *

Copy and paste relevant sections from the manuscript (include quotes in quotation marks "like this" to indicate direct quotes from your manuscript), or elaborate on this item by providing additional information not in the ms, or briefly explain why the item is not applicable/relevant for your study

"Of the 21 patients in the VR group, no subject reported problems with motion sickness or associated nausea; also, no treatment had to be stopped due to motion sickness or nausea. This is congruent with the results of our feasibility trial with stroke patients and healthy older adults, where we demonstrated that applying specific principles in VR development, e.g., having complete control of all motion in VR, reduces the risk of suffering motion sickness and is feasible for use in special needs groups like stroke patients [16]. Patients in the control group, using CCT, reported no negative side effects as well. Patients conducted, on average, 18.7 successful treatment sessions."

19-i) Include privacy breaches, technical problems

Include privacy breaches, technical problems. This does not only include physical “harm” to participants, but also incidents such as perceived or real privacy breaches [1], technical problems, and other unexpected/unintended incidents. “Unintended effects” also includes unintended positive effects [2].

subitem not at all important

1 ○

2 ○

3 ○

4 ○

5 ○

essential

Does your paper address subitem 19-i?

Copy and paste relevant sections from the manuscript (include quotes in quotation marks "like this" to indicate direct quotes from your manuscript), or elaborate on this item by providing additional information not in the ms, or briefly explain why the item is not applicable/relevant for your study

Meine Antwort

19-ii) Include qualitative feedback from participants or observations from staff/researchers

Include qualitative feedback from participants or observations from staff/researchers, if available, on strengths and shortcomings of the application, especially if they point to unintended/unexpected effects or uses. This includes (if available) reasons for why people did or did not use the application as intended by the developers.

subitem not at all important

1 ○

2 ○

3 ○

4 ○

5 ○

essential

Does your paper address subitem 19-ii?

Copy and paste relevant sections from the manuscript (include quotes in quotation marks "like this" to indicate direct quotes from your manuscript), or elaborate on this item by providing additional information not in the ms, or briefly explain why the item is not applicable/relevant for your study

Meine Antwort

DISCUSSION

22) Interpretation consistent with results, balancing benefits and harms, and considering other relevant evidence

NPT: In addition, take into account the choice of the comparator, lack of or partial blinding, and unequal expertise of care providers or centers in each group

22-i) Restate study questions and summarize the answers suggested by the data, starting with primary outcomes and process outcomes (use)

Restate study questions and summarize the answers suggested by the data, starting with primary outcomes and process outcomes (use).

subitem not at all important

1 ◯

2 ◯

3 ◯

4 ◯

5 ◯

essential

Does your paper address subitem 22-i? *

Copy and paste relevant sections from the manuscript (include quotes in quotation marks "like this" to indicate direct quotes from your manuscript), or elaborate on this item by providing additional information not in the ms, or briefly explain why the item is not applicable/relevant for your study

For a distinct description of subitem 22-i see chapter Discussion which includes all required items.

22-ii) Highlight unanswered new questions, suggest future research

Highlight unanswered new questions, suggest future research.

subitem not at all important

1

2

3

4

5

essential

Does your paper address subitem 22-ii?

Copy and paste relevant sections from the manuscript (include quotes in quotation marks "like this" to indicate direct quotes from your manuscript), or elaborate on this item by providing additional information not in the ms, or briefly explain why the item is not applicable/relevant for your study

Meine Antwort

20) Trial limitations, addressing sources of potential bias, imprecision, and, if relevant, multiplicity of analyses

### 20-i) Typical limitations in ehealth trials

Typical limitations in ehealth trials: Participants in ehealth trials are rarely blinded. Ehealth trials often look at a multiplicity of outcomes, increasing risk for a Type I error. Discuss biases due to non-use of the intervention/usability issues, biases through informed consent procedures, unexpected events.

subitem not at all important

1 ◯

2 ◯

3 ◯

4 ◯

5 ◯

essential

### Does your paper address subitem 20-i? *

Copy and paste relevant sections from the manuscript (include quotes in quotation marks "like this" to indicate direct quotes from your manuscript), or elaborate on this item by providing additional information not in the ms, or briefly explain why the item is not applicable/relevant for your study

"Although this study provides valuable insights into the impact of immersive VR on cognitive rehabilitation, many questions remain unanswered. Specifically, we identified the following limitations that should be considered: Future research in this field should be conducted with larger sample groups. This randomized controlled pilot study builds a profound understanding of the potential immersive VR can have in neurological rehabilitation for cognitive impairment in patients with MMST>20, affected by stroke, to the largest extent. Conclusions cannot be drawn to patients below this cutoff score. We also need more information on the manifestation of training effects, e.g., if the measured results are still the same 6-12 months after the intervention and if an actual transfer effect to ADLs occurs. Long-term studies with follow-ups of multiple years are necessary.

The applied VR environment was limited to only two training scenarios inspired by ADLs. More training scenarios replicating other ADLs should be developed and tested for future research.

Even though there are findings about the application of VR in rehabilitation in other patient groups, e.g., multiple sclerosis, psychiatric disorders, and pain management [53], the here-used application has chiefly been tested in the rehabilitation of patients with acute brain affection. Therefore, the evaluation needs to consider patients from the abovementioned groups to draw further conclusions in other patient groups."

## 21) Generalisability (external validity, applicability) of the trial findings

NPT: External validity of the trial findings according to the intervention, comparators, patients, and care providers or centers involved in the trial

## 21-i) Generalizability to other populations

Generalizability to other populations: In particular, discuss generalizability to a general Internet population, outside of a RCT setting, and general patient population, including applicability of the study results for other organizations

subitem not at all important

1 ○

2 ○

3 ○

4 ○

5 ○

essential

## Does your paper address subitem 21-i?

Copy and paste relevant sections from the manuscript (include quotes in quotation marks "like this" to indicate direct quotes from your manuscript), or elaborate on this item by providing additional information not in the ms, or briefly explain why the item is not applicable/relevant for your study

Meine Antwort

21-ii) Discuss if there were elements in the RCT that would be different in a routine application setting

Discuss if there were elements in the RCT that would be different in a routine application setting (e.g., prompts/reminders, more human involvement, training sessions or other co-interventions) and what impact the omission of these elements could have on use, adoption, or outcomes if the intervention is applied outside of a RCT setting.

subitem not at all important

1 ○

2 ○

3 ○

4 ○

5 ○

essential

Does your paper address subitem 21-ii?

Copy and paste relevant sections from the manuscript (include quotes in quotation marks "like this" to indicate direct quotes from your manuscript), or elaborate on this item by providing additional information not in the ms, or briefly explain why the item is not applicable/relevant for your study

Meine Antwort

OTHER INFORMATION

23) Registration number and name of trial registry

Does your paper address CONSORT subitem 23? *

Copy and paste relevant sections from the manuscript (include quotes in quotation marks "like this" to indicate direct quotes from your manuscript), or elaborate on this item by providing additional information not in the ms, or briefly explain why the item is not applicable/relevant for your study

"The trial was registered at the federal register for clinical trials in Germany DRKS under the file number DRKS000023605 and at the central European register for clinical research with medical devices EUDAMED in line with the medical device regulation MDR under the file number CIV-20-06-033349."

24) Where the full trial protocol can be accessed, if available

Does your paper address CONSORT subitem 24? *

Cite a Multimedia Appendix, other reference, or copy and paste relevant sections from the manuscript (include quotes in quotation marks "like this" to indicate direct quotes from your manuscript), or elaborate on this item by providing additional information not in the ms, or briefly explain why the item is not applicable/relevant for your study

The protocol is deposited in the German Register of Clinical Trials.

25) Sources of funding and other support (such as supply of drugs), role of funders

Does your paper address CONSORT subitem 25? *

Copy and paste relevant sections from the manuscript (include quotes in quotation marks "like this" to indicate direct quotes from your manuscript), or elaborate on this item by providing additional information not in the ms, or briefly explain why the item is not applicable/relevant for your study

"The Federal State of Baden-Württemberg supported this work through the Innovationsgutschein BW High-Tech Startup funding program."
"The co-authors of this publication, Barbara Stegmann and Julian Specht are the co-founders of living brain."

X27) Conflicts of Interest (not a CONSORT item)

X27-i) State the relation of the study team towards the system being evaluated

In addition to the usual declaration of interests (financial or otherwise), also state the relation of the study team towards the system being evaluated, i.e., state if the authors/evaluators are distinct from or identical with the developers/sponsors of the intervention.

subitem not at all important

1 ○

2 ○

3 ○

4 ○

5 ○

essential

Does your paper address subitem X27-i?

Copy and paste relevant sections from the manuscript (include quotes in quotation marks "like this" to indicate direct quotes from your manuscript), or elaborate on this item by providing additional information not in the ms, or briefly explain why the item is not applicable/relevant for your study

Meine Antwort

About the CONSORT EHEALTH checklist

As a result of using this checklist, did you make changes in your manuscript? *

- ○ yes, major changes
- ◉ yes, minor changes
- ○ no

What were the most important changes you made as a result of using this checklist?

I added minor things to the manuscript.

How much time did you spend on going through the checklist INCLUDING making changes in your manuscript *

I spent about 5 hours going through the checklist and making changes to the manuscript

As a result of using this checklist, do you think your manuscript has improved? *

- ◉ yes
- ○ no
- ○ Sonstiges:

Would you like to become involved in the CONSORT EHEALTH group?

This would involve for example becoming involved in participating in a workshop and writing an "Explanation and Elaboration" document

- ○ yes
- ○ no
- ○ Sonstiges:

Any other comments or questions on CONSORT EHEALTH

Meine Antwort

STOP - Save this form as PDF before you click submit

To generate a record that you filled in this form, we recommend to generate a PDF of this page (on a Mac, simply select "print" and then select "print as PDF") before you submit it.

When you submit your (revised) paper to JMIR, please upload the PDF as supplementary file.

Don't worry if some text in the textboxes is cut off, as we still have the complete information in our database. Thank you!

Final step: Click submit !

Click submit so we have your answers in our database!

Senden

Alle Eingaben löschen

Geben Sie niemals Passwörter über Google Formulare weiter.

Dieses Formular wurde außerhalb Ihrer Domain erstellt. Missbrauch melden - Nutzungsbedingungen - Datenschutzerklärung

Google Formulare
